# Potential Clinical Application of Analysis of Bisphenols in Pericardial Fluid from Patients with Coronary Artery Disease with the Use of Liquid Chromatography Combined with Fluorescence Detection and Triple Quadrupole Mass Spectrometry

**DOI:** 10.3390/molecules30010140

**Published:** 2025-01-01

**Authors:** Tomasz Tuzimski, Szymon Szubartowski, Janusz Stążka, Kamil Baczewski, Daria Janiszewska, Viorica Railean, Bogusław Buszewski, Małgorzata Szultka-Młyńska

**Affiliations:** 1 Department of Physical Chemistry, Faculty of Pharmacy, Medical University of Lublin, Chodźki 4a, 20-093 Lublin, Poland; szymon.szubartowski95@gmail.com; 2 Doctoral School of Medical University of Lublin, Medical University of Lublin, Chodźki 6, 20-093 Lublin, Poland; 3 Department of Cardiac Surgery, Medical University of Lublin, Jaczewskiego 8 (USK Nr 4), 20-093 Lublin, Poland; janusz.stazka@umlub.pl (J.S.); kamil.baczewski@umlub.pl (K.B.); 4 Department of Environmental Chemistry and Bioanalytics, Gagarina 7, Faculty of Chemistry, Nicolaus Copernicus University, 87-100 Torun, Poland; janiszewska_daria@doktorant.umk.pl (D.J.); bbusz@chem.umk.pl (B.B.); mszultka@umk.pl (M.S.-M.); 5 Department of Infectious, Invasive Diseases and Veterinary Administration, Institute of Veterinary Medicine, Nicolaus Copernicus University in Torun, Gagarina 7, 87-100 Torun, Poland; viorica.railean@umk.pl; 6 Centre for Modern Interdisciplinary Technologies, Nicolaus Copernicus University, Wilenska 4, 87-100 Torun, Poland; 7 Professor Jan Czochralski Kuyavian-Pomeranian Scientific Technological Centre, Krasinskiego 4, 87-100 Torun, Poland

**Keywords:** dispersive liquid–liquid microextraction (DLLME), LC–ESI–QqQ, pericardial fluid, bisphenol, bisphenol A diglicydyl ether (BADGE) with analogs

## Abstract

Bisphenols may negatively impact human health. In this study, we propose the use of HPLC–FLD for the simultaneous determination of bisphenols in pericardial fluid samples collected from patients with coronary artery disease undergoing coronary artery bypass surgery. For sample preparation, a fast, simple, and ”green” DLLME method was used, achieving mean recovery values in the range of 62%–98% with relative standard deviations between 2% and 6% for all analytes. Quantitative analysis of bisphenols in the samples was then performed by LC–MS/MS on a triple quadrupole (QqQ) mass spectrometer and electrospray ionization (ESI-/ESI+) was applied in the negative and positive ion modes, respectively. The LODs and LOQs ranged from 0.04 ng/mL to 0.37 ng/mL and 0.12 ng/mL to 1.11 ng/mL, respectively. Pericardial fluid was collected from patients with coronary artery disease during coronary artery bypass surgery. Bisphenol residues were identified and quantified in samples from 19 patients. The procedure was successfully applied to the biomonitoring of free forms of 14 bisphenols in pericardial fluid. After statistical examination of the relationships between the selected variables, a strongly positive correlation was found between creatinine kinase and troponin I, as well as the number of venous anastomoses, circulation time, and clamp cap time.

## 1. Introduction

Under normal conditions, physiologic serous fluid exists within the pericardial sac and acts as lubrication for the heart. A pericardial effusion occurs when this fluid exceeds 15–50 mL [1,2,3]. Pericardial tamponade refers to hemodynamic instability and clinical symptoms directly resulting from this effusion. This occurs when the pressure exerted on one or more cardiac chambers by the pericardial effusion (i.e., intrapericardial pressure) exceeds the pressures within the cardiac chambers (i.e., intracardiac pressure), leading to the obstruction of normal chamber filling [2].

The human heart’s pericardium has received increasing attention in recent years due to the growing interest in epicardial approaches for cardiac interventions, particularly in treating cardiac arrhythmias that are refractory to conventional endocardial approaches [4]. In 1996, Sosa et al. first introduced the epicardial approach for the treatment of refractory ventricular tachycardia [5]; further accumulating experience [4,6] has resulted in the technique becoming well established [2,7]. Currently, the epicardial approach is applied across a wide range of indications [4,8], not only for treating refractory ventricular arrhythmias but also for managing supraventricular arrhythmias [4,9], with various epicardial devices used to support the ablation procedure [4,10]. 

Bisphenols (BPs) are chemicals that are widely used in the production of everyday products. They can be found in food contact materials, thermal paper, toys, and much more [11,12]. Unfortunately, they may negatively impact human health [13], and due to their toxicity, some restrictions have been placed on their use. For example, the European Food Safety Authority set a Specific Migration Limit (SML) for bisphenol A (BPA) at 0.6 mg per kg of food (Commission Regulation (EU) No 10/2011), later updated to 0.05 mg per kg of food (Commission Regulation (EU) No 213/2018). Moreover, BPA has been prohibited in baby feeding bottles since 2011 (Commission Regulation (EU) No 8/2011). Only a few other BPs are currently under no restriction on their use. Bisphenol S (BPS) has an SML of 0.05 mg per kg of food (Commission Regulation (EU) No 10/2011) [14]. This ongoing concern about BP exposure underscores the need for continuous biomonitoring of these chemicals in environmental and biological samples [14].

Human exposure to xenobiotics (e.g., bisphenols) is widespread, with exposure doses ranging from several hundred nanograms (ng) to a few micrograms (μg) per kilogram (kg) of body weight (bw) per day (d) through diet and <10 ng/kg bw/d via inhalation of indoor air and ingestion of indoor dust [15,16]. BPs have been detected in various human samples, including urine [17,18,19], human breast milk [15,20,21,22,23], blood or serum samples [24,25,26], and amniotic fluid [26,27,28]. 

Pericardial fluid samples may represent an additional human sample used in the biomonitoring of BPs. 

Current methods for determining bisphenols in biological samples predominantly rely on chromatographic techniques such as LC with fluorescence (LC–FLD), diode-array (LC–DAD), and MS detection (LC–MS, LC–MS/MS or UHPLC-MS/MS) [17,18,19,20,21,22,23,25,27,28,29]. Chotoye et al. introduced the application of multiple heart-cutting two-dimensional liquid chromatography (2D-LC) to reproducibly identify and quantify bisphenols in canned food items [30].

Rancière et al. [31] published a study reviewing the epidemiological literature on the relation between BPA exposure and the risk of cardiometabolic disorders. The authors included observational studies (cohort, case–control, and cross-sectional studies) conducted in children or adults, measuring urinary BPA (uBPA) [31]. 

An interesting alternative to formerly used sample preparation techniques is dispersive liquid–liquid microextraction (DLLME), which was originally developed in 2006 for isolating polyaromatic hydrocarbons from water samples [32]. DLLME has also been successfully applied in the biomonitoring of BPs in human breast milk samples by F. Vela-Soria et al. [22] with the use of LC–MS/MS. 

In forensic toxicology, body fluids are important materials not only as alternatives to blood but also for the investigation of postmortem drug redistribution and pharmaco-kinetic and toxicokinetic analysis. In the studies published, pericardial fluid has been proposed as a specimen for toxicological analysis [33,34,35,36]. The authors analyzed data where drugs from the following groups were detected: psychostimulants, narcotic/antitussive analgesics, barbiturates, phenothiazine derivatives, benzodiazepines, other sedative/hypnotics, anesthetics, and others [35], intravenously or orally abused methamphetamine and its possible metabolite, amphetamine [35], and cocaine and its metabolites (benzoylecgonine and cocaethylene) [36]. The data confirmed that pericardial fluid can be used for the analysis of selected medicines and drugs as an alternative biological sample in forensic toxicology. This is particularly useful when the procurement of blood samples is difficult or as an additional biological sample.

Pericardial effusions can indicate serious systemic pathology and the underlying diagnosis may be evident at the time of detection, such as in the presence of active autoimmune disease. To distinguish transudative from exudative effusions, paired serum and pericardial fluid protein, and lactate dehydrogenase (LDH) levels are examined. However, unlike cytology or microbiology, which can directly identify the cause, this biochemical strategy is limited as it only narrows the differential diagnoses. The 2015 European Society of Cardiology guidelines on the diagnosis and management of pericardial disease recommend assessing paired pericardial fluid and serum protein and lactate dehydrogenase (LDH) levels to determine if the pericardial fluid is an exudate or a transudate.

However, our understanding of the normal biochemical composition of pericardial fluid remains limited, and there are no established reference ranges. While Light’s criteria are often applied, this practice is questionable because they were specifically developed for pleural fluid assessment. Moreover, a recent study on physiologic pericardial fluid revealed that most samples were classified as exudates due to high physiologic fluid protein and lactate dehydrogenase (LDH) levels. Although some studies have reported elevated fluid lactate dehydrogenase (LDH) levels in malignant effusions, lactate dehydrogenase (LDH) levels alone are insufficient to accurately differentiate between malignant and nonmalignant effusions. 

Therefore, in another publication [37], the authors aimed to examine the diagnostic yield of pericardial fluid biochemistry and cytology, as well as their prognostic significance in patients with percutaneously drained pericardial effusions, both with and without malignancy. Patients (n = 179) were grouped based on the presence or absence of underlying malignancy. The study found no significant differences in pericardial fluid protein and lactate dehydrogenase between the two groups. However, the diagnostic yield from pericardial fluid analysis was notably higher in the malignant group; approximately 75% of newly diagnosed malignancies had positive fluid cytology. The authors concluded that pericardial fluid biochemistry has limited value in determining the etiology of pericardial effusions; instead, fluid cytology is the most important diagnostic test and, therefore, novel diagnostic tests for pericardial diseases should be developed [37].

B-type natriuretic peptide (BNP) is released by ventricular muscles and plays a role in preventing their fibrosis. The concentration of BNP or its precursor, NT-proBNP, in plasma increases in patients with heart failure. For this reason, among others, measuring the levels of these peptides is crucial in the treatment of patients with heart failure. 

Of course, if the amount of oxygen supplied is below the critical threshold, cell death occurs, indicated by increased markers of heart damage in the blood, such as troponin and cardiac enzymes, particularly the cardiac isoenzyme creatine kinase. These elevated markers of heart damage lead to characteristic changes in ECG recording.

Aortic valve stenosis (AS), aortic stenosis, is a heart defect characterized by the narrowing of the left ventricular outflow tract, reducing the area of the aortic valve opening to the extent that it impedes blood flow from the left ventricle to the aorta. AS is the third most common heart disease in developed countries, after hypertension and ischemic heart disease. In the examined cohort, the group consisted of patients diagnosed with aortic stenosis. 

The study by Fellahi et al. assessed the clinical relevance of using pericardial cTnI to evaluate perioperative myocardial damage in symptomatic patients undergoing elective CABG surgery. The study included 102 subjects with symptomatic coronary disease [38]. Postoperative arterial and pericardial blood samples were collected, and the patients were allocated into one of three groups based on 12-lead electrocardiogram (ECG) abnormalities observed during the first 24 h post-surgery: normal ECG (group 1); nonspecific ECG abnormalities (group 2); and perioperative Q- wave MI (group 3) [38]. Peak pericardial CTnI concentrations were much higher than peak serum concentrations across all subjects and significantly greater in group 3 compared to groups 1 and 2 (1318 ± 1810 ng/mL vs. 367 ± 339 ng/mL and 558 ± 608 ng/mL, respectively; *p* < 0.01). However, there was no significant difference between the groups regarding pericardial/serum CTnI ratios, indicating that the time courses of CTnI were similar in both pericardial fluid and serum. A significant correlation was observed between serum and pericardial CTnI concentrations (R = 0.70, *p* < 0.001). Moreover, receiver operator characteristic curves were plotted, and the area under the curve demonstrated a lack of accuracy in predicting perioperative MIs and an increase in serum cTnI. Additionally, peak and early pericardial CTnI were not reliable in predicting an increase in serum CTnI beyond a cutoff value of 19 ng/mL [38].

A similar study was conducted by Cihan et al. to evaluate the diagnostic potential of pericardial cTnI for detecting postoperative MI [39]. The study included 64 subjects undergoing elective CABG surgery, who were allocated to one of two groups based on 12-lead electrocardiogram (ECG) abnormalities observed during the first 24 h post-surgery: normal and non-specific ECG (group 1) and perioperative Q-wave MI (group 2). Pericardial concentrations were higher than serum concentrations in all subjects during the first 24 h post-surgery. On the other hand, a greater than two-fold increase in the pericardial/serum MG ratio was observed in all patients who experienced perioperative Q-wave MI, with the lowest ratio being 2.75. In contrast, only 1 of the 59 patients in group 1 had this ratio greater than 2, with the highest value being 2.15 at the time of admission to the ICU [39].

Tambara et al. described a study to measure troponin T (TnT) concentrations in serum and PF in 34 patients undergoing CABG for either unstable angina or stable coronary artery disease [40]. Patients were divided into two groups: group 1 (n=17) had angina symptoms and/or ST-changes in ECG monitoring within 24 h before the operation; while group 2 (n=17) included those without these symptoms or changes. Serum heart-type cytoplasmic fatty acid-binding protein levels were slightly elevated in both groups, compared to their normal values. No significant difference was found in TnT concentrations between pericardial fluid and serum [40].

In another study [41], blood and pericardial fluid samples were collected from 31 patients at the time of surgery and postoperatively, from 4 to 48 h after undergoing coronary artery bypass grafting, valve replacement, or valve repair (mitral or aortic). Kramer et al. [41] investigated whether cardiac surgery resulted in the release of pro-oxidant and pro-inflammatory molecules into the pericardial fluid and found that the levels of cTnI were significantly higher in pericardial fluid compared to serum. Additionally, elevated pericardial fluid cTnI levels were associated with increased markers of oxidative stress. Furthermore, plasma cTnI levels remained exceptionally low throughout the postoperative period, while pericardial fluid levels were elevated 50 h post-surgery, with a sudden spike around 10 h postoperative. The authors concluded that postoperative pericardial fluid presents a highly pro-oxidant environment following cardiac surgery, with elevated levels of cardiac injury markers [41].

In another study by Butts et al. [42], inflammation in the pericardial fluid following cardiac surgery was examined in 19 patients who underwent CABG, a CABG and valve procedure, or a valve procedure alone. The study found that cTnI levels were markedly elevated in the pericardial fluid after surgery. Moreover, cTnI levels in the pericardial fluid were significantly higher at 48 h in individuals who developed POAF, compared to those who did not. When compared to the blood, the pericardial fluid concentration of cTnI was higher by orders of magnitude [42].

To the best of our knowledge, this study is the first to examine bisphenol residues in pericardial fluid collected from Polish patients with coronary artery disease undergoing coronary artery bypass surgery. The manuscript also aims to link the frequency of bisphenols and their concentrations in pericardial fluid collected during coronary artery bypass surgery, and an attempt was made to correlate of frequency/concentrations of bisphenols with some popular causes of cardiovascular (CV) system disease. Despite very invasive sampling, the data obtained during analysis may improve current knowledge about the toxicity of these analytes on the human cardiovascular system (e.g., in the pericardial fluid collected from patients diagnosed with aortic stenosis).

## 2. Results and Discussion

### 2.1. Analysis of Bisphenols with the Use of HPLC–FLD

**Figure 1:**
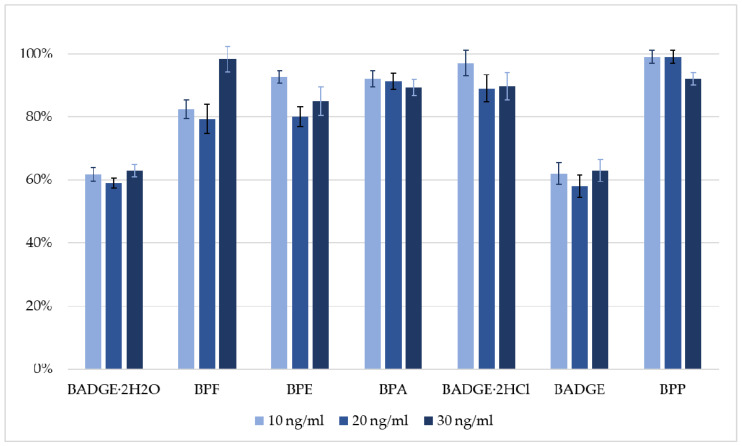
The diagram shows the recovery values for the analyzed bisphenols with relative standard deviations between 2% and 6% for all analytes.

In previous studies [20,28], preliminary experiments were conducted to optimize the DLLME-based extraction conditions for bisphenols from biological samples, specifically human breast milk and amniotic fluid. This optimization involved adjusting the types, volumes, and ratios of solvents used. All experiments were performed using the well-characterized DLLME-based extraction procedure as described in a previous study by Szubartowski and Tuzimski [20]. The flowchart of the final procedure is presented in the Materials and Methods section. The DLLME-based extraction procedure demonstrated strong performance across all analytes, with recovery rates ranging from 62 to 98% and relative standard deviations (RSD) below 6% (Figure 1). 

**Figure 2:**
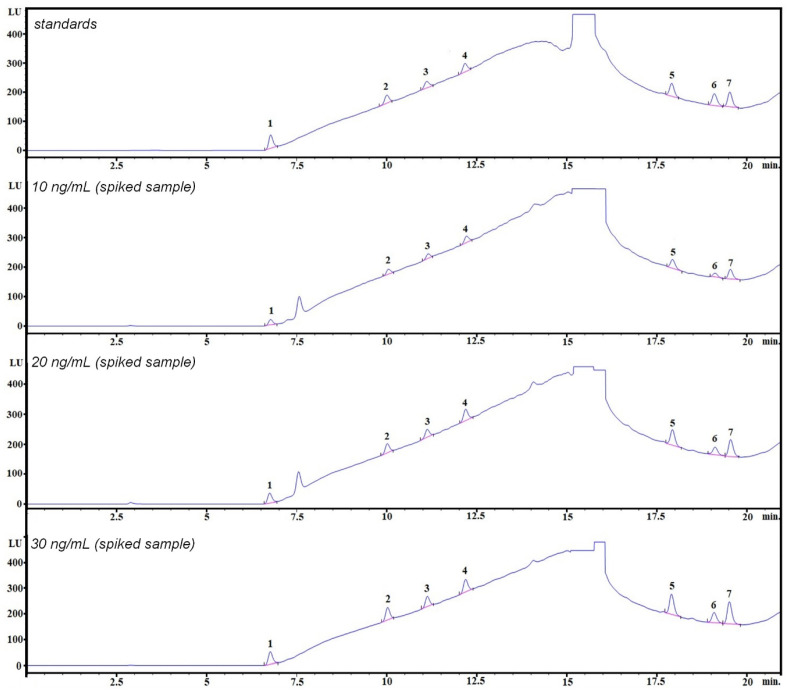
Example chromatograms obtained during the HPLC–FLD analysis: mixture of bisphenol standards (25 ng/mL) and three spiked samples at 10 ng/mL, 20 ng/mL, and 30 ng/mL. 1—BADGE∙2H_2_O, 2—BPF, 3—BPE, 4—BPA, 5—BADGE∙2HCl, 6—BADGE, 7—BPP.

LC coupled with detection techniques (DAD, FLD) has shown significant potential for the simultaneous determination of bisphenols, valued for its simplicity, rapidity, high efficiency, and low cost [30]. This study presents a cost-effective and sensitive liquid chromatography fluorescence detector (HPLC–FLD) method for the simultaneous determination of seven bisphenols in pericardial fluid samples. The exemplary chromatogram of the mixture of bisphenol standards in acetonitrile (ACN): water 1:1 (*v*/*v*) is shown in Figure 2 (top). The chromatographic conditions were based on a previously published method [20] with only slight changes. The details are shown in the Materials and Methods section. The separation of the seven analytes under investigation is possible by applying a gradient elution mode in less than 20 min, obtaining satisfactory selectivity, efficiency, and peak symmetry for all the bisphenols. In the detection techniques, the absorption of analytes is often monitored at 225–240 nm, and the emission at 300–315 nm, depending on the bisphenol analyzed. The excitation wavelength of 240 nm and an emission wavelength of 300 nm with signal amplification set at 15 were found as the most optimal variation in the operation of the fluorescence detector, taking into account the signal strength of analytes and the noise level. The method successfully separated bisphenols from the remaining matrix components (Figure 2). No significant matrix effect was observed for all examined analytes (please see Supplementary Materials, Figure S1). 

The selectivity evaluation was based on comparing the chromatograms from the averaged human pericardial fluid sample blank, the spiked human pericardial fluid sample before the extracting procedure, and the spiked human pericardial fluid after the procedure. Identification of bisphenols was based on comparing the retention times in the sample and in the mixture of bisphenols standards.

Recovery studies were conducted for spiked samples at three different concentration levels: 10 ng mL^−1^; 20 ng mL^−1^, and 30 ng mL^−1^ in an averaged pericardial fluid collected from patients with coronary artery disease undergoing coronary artery bypass surgery. Concentration levels were chosen based on the highest LOQ values, with 10 ng/mL, 20 ng/mL, and 30 ng/mL were approximately 1.5xLOQ, 3xLOQ, and 4.5xLOQ, respectively. Unfortunately, during the assessment of recovery values, due to the limited amount of pericardial fluid, it was not possible to perform analyses for pericardial fluid samples spiked at higher concentration levels.

In order to conduct the most reliable studies, during the subsequent stages of the experiments, we prepared samples of the average pericardial fluid matrix on an ongoing basis. The average pericardial fluid matrix was prepared by mixing several samples taken from 5 to 7 patients. The chromatogram at the 30 ng/mL enrichment level corresponds to the chromatogram for the average pericardial fluid matrix taken from a different group of patients than the one used for the 10 ng/mL or 20 ng/mL matrix. This accounts for the difference between the two chromatograms. It would be ideal if commercially available reference pericardial fluid matrices existed. However, there are no reference pericardial fluid matrices, such as, e.g., urine. Since such matrices are unavailable, small differences (such as the additional peak at 7.5 min) arise in the average pericardial fluid matrix. The peak at 7.5 min is attributed to a matrix component and is well separated from the other analytes.

Average recovery values were obtained by six replicates per day during the next three days (intra- and inter-day validation). The results of this experiment are shown in Table S2. Additionally, results for spiked samples at 25 ng mL^−1^ are also shown in Table S3 in the Supplementary Materials.

The optimized extraction procedure demonstrated strong analytical performance in terms of extraction efficiency and repeatability. Average values of recoveries were in the ranges of 61–99%, 58–92%, and 63–98% at the following three spiking levels: 10 ng mL^−1^; 20 ng mL^−1^, and 30 ng mL^−1^, respectively. In all cases, relative standard deviations expressed as percentages (RSD%) were below 6% (n = 9). Additionally, as shown in Figure 2, no significant matrix effect was observed at all three spiking levels (from 10 ng mL^−1^ to 30 ng mL^−1^). No significant matrix effect was observed at all spiking examined levels, with ME% values ranging from 0 to 10%. These results confirm the reliability of the recovery studies of the proposed procedure. The chromatogram of the pericardial fluid matrix (Figure S1) does not indicate any significant influence of its other interferences on the quantification of the analytes and their recoveries from the matrix (e.g., matrix suppression).

However, to enhance the selectivity when analyzing bisphenols at low concentrations in matrices, for the correct identification of analytes, MS/MS with a QqQ analyzer is the preferred choice. Detection methods in LC-based procedures rely mostly on triple quadrupole tandem mass spectrometry (QqQ–MS/MS) operating in the multiple reaction monitoring (MRM) mode, which enables the accurate identification and quantification of targeted analytes. 

Consequently, in the next phase of the study, the fast, cheap, and ‘green’ DLLME sample preparation technique was applied before quantitative analysis with the use of high-performance liquid chromatography coupled with triple quadrupole-tandem mass spectrometry (QqQ–MS/MS).

### 2.2. Analysis of Bisphenols by LC–MS/MS in Pericardial Fluids Samples

Pericardial fluid was collected from 19 patients with coronary artery disease undergoing coronary artery bypass surgery. In this study, a method based on dispersive liquid–liquid microextraction (DLLME) combined with LC–MS/MS analysis was developed for the determination of 14 bisphenols in the pericardial fluid samples. 

In the present study, bisphenols were identified and quantified in samples from 19 patients (please see Supplementary Materials Table S1). 

In the quantitative analysis, the concentrations ranges of bisphenols were as follows: for BPA from 0.35 ng/mL to 3.63 ng/mL (n = 19), bisphenol F (BPF) from 0.67 ng/mL to 1.79 ng/mL (n = 10), bisphenol E (BPE) from 0.73 ng/mL to 1.84 ng/mL (n = 19), BADGE from 0.14 ng/mL to 1.45 ng/mL (n = 19), and BADGE•2H_2_O from 1.24 ng/mL to 1.31 ng/mL (n = 4). 

Bisphenols were also identified below their LOQs values (<LOQ) in 40 total samples: BPF (n = 5) BPP (n = 12), BADGE•2H_2_O (n = 11), and BADGE•2HCl (n = 12). BPS, BPAF, and BADGE•H_2_O were not identified in any samples.

Other analytes were also identified and quantified during LC–ESI–QqQ analyses: bisphenol Z (BPZ), biphenol B (BPB), bisphenol AP (BPAP), and BADGE•H_2_O•HCl. In the quantitative analysis, the concentration ranges of these bisphenols were as follows: BPZ from 0.58 ng/mL to 0.68 ng/mL (n = 7), BPB from 0.68 ng/mL to 0.72 ng/mL (n = 2), BPAP from 0.25 ng/mL to 1.93 ng/mL (n = 16), and BADGE•H_2_O•HCl from 0.67 ng/mL to 1.79 ng/mL (n = 9). There analytes were also identified below their LOQs values (<LOQ) in 33 total samples: BPZ (n = 10), BPB (n = 13), BPAP (n = 3), and BADGE•H_2_O•HCl (n = 7).

**Figure 3:**
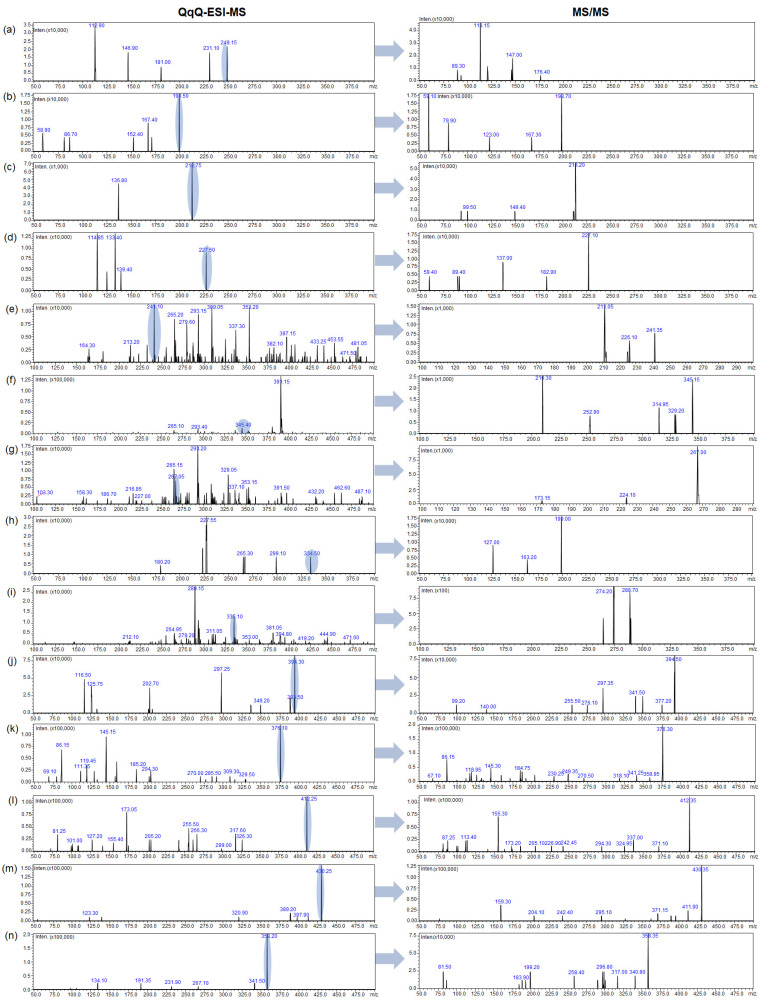
QqQ–ESI–MS and MS/MS spectra of following bisphenols residues detected in pericardial fluid samples: (**a**) BPS (*m*/*z* = 249), (**b**) BPF (*m*/*z* = 199), (**c**) BPE (*m*/*z* = 213), (**d**) BPA (*m*/*z* = 227), (**e**) BPB (*m*/*z* = 241), (**f**) BPP (*m*/*z* = 345), (**g**) BPZ (*m*/*z* = 267), (**h**) BPAF (*m*/*z* = 335), (**i**) BPAP (*m*/*z* = 335), (**j**) BADGE•2H_2_O (*m*/*z* = 394), (**k**) BADGE•H_2_O (*m*/*z* = 376), (**l**) BADGE•H_2_O•HCl (*m*/*z* = 412), (**m**) BADGE•2HCl (*m*/*z* = 430), and (**n**) BADGE (*m*/*z* = 358).

The LC–ESI–QqQ–MS/MS method was applied for the determination and identification of studied bisphenols in pericardial fluid samples. MS/MS experiments were performed in the QqQ mass spectrometer (8050 Shimadzu, Kyoto, Japan) equipped with an electrospray ionization (ESI) source. After determining the optimal conditions for isolating the precursor ion (analyte proton adduct), full scan MS/MS mode was used to record product ions from the standard solution of each target compound. The fragmentation amplitude and isolation width for each analyte were manually optimized to enhance the method’s selectivity and sensitivity, and to select the most intense and characteristic fragmentation ions for qualitative analysis and one of the highest intensity for quantitative analysis. The identified bisphenols were further characterized based on the MS/MS fragmentation patterns (Figure 3, please see also Supplementary Materials Figure S2).

The present study aimed to evaluate the association between the types and concentrations of bisphenols in pericardial fluid samples collected from patients with coronary artery disease undergoing coronary artery bypass surgery, as well as chronic cardiometabolic disorders (significantly overweight, obesity (BMI), CVD, and hypertension).

**Figure 4:**
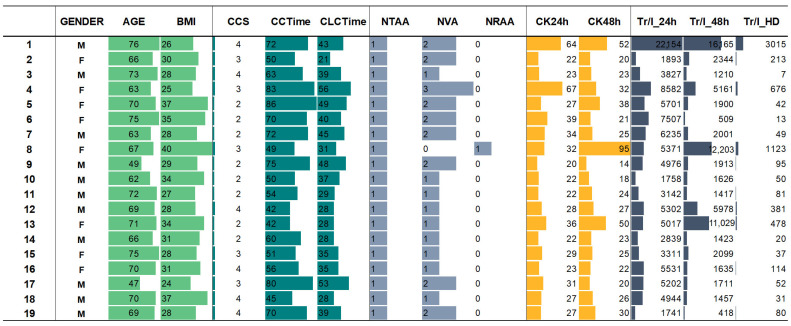
Selected clinical data of 19 patients with coronary artery diseases and undergoing coronary artery bypass surgery.

Selected clinical data of 19 patients with coronary artery disease undergoing coronary artery bypass surgery are presented in Figure 4.

Abbreviations: Gender M/F: Male/Female; CCS/Central cord syndrome; CCTime/circulation time (min); CLCTime/clamp cap time (min); NTAA/no of thoracic artery anastomoses; NVA/no of venous anastomoses; NRAA/no of radial artery anastomoses; CK24h/creatinine kinase (U/L), 24 h after the surgency; CK48h/creatinine kinase (U/L), 48 h after the surgency; Tr/I_24 h/Troponin I (ng/L), 24 h after the surgency; Tr/I_48 h/Troponin I (ng/L), 48 h after the surgency; Tr/I_HD/Troponin I (ng/L), Hospital discharge data.

Surgency operation data: CCS, CLCTime, NTAA, NVA, NRAA; First 24 h (to 24 h after the surgency): CK24h; Tr/I_24h; Second 24 h (to 48 h after the surgency): CK48h; Tr/I_48h; Hospital discharge data: Tr/I_HD.

### 2.3. Statistical Analysis

**Figure 5:**
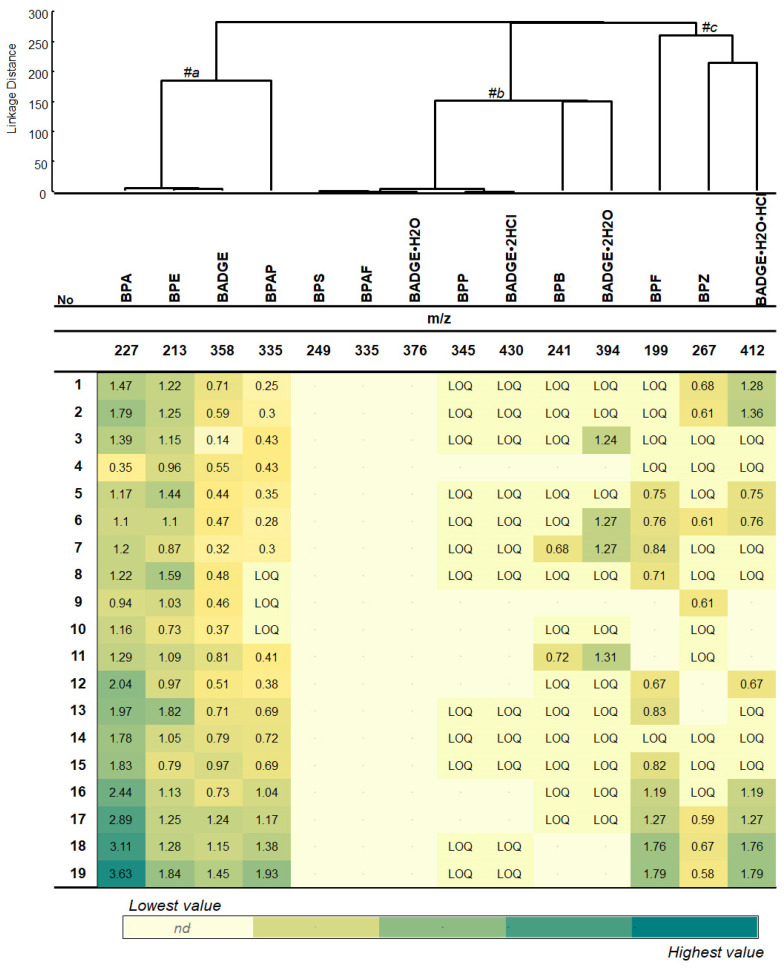
Heatmap showing the distribution and concentration (ng/mL) of quantified bisphenols in pericardial fluids collected from 19 patients with coronary artery diseases and undergoing coronary artery bypass surgery. The samples numbers (1–19) indicate the name of samples analyzed by LC–ESI–QqQ. Hierarchical cluster analysis shows the correlations between the analyzed bisphenols. #a, #b, #c—indicates the main formed clusters.

Figure 5 illustrates the abundance of bisphenols in the pericardial fluid samples collected from 19 patients. The heat map provides a snapshot of the quantified concentrations of bisphenols. 

The hierarchical clustering model revealed the formation of three main clusters: BPA, BPE, BADGE, and BPAP were found in all investigated samples except for samples 8, 9, and 10 (#a); the group of BPS, BPAF, BADGE•H_2_O, BADGE•2HCl, and BPP were not detected in all samples or detected at the LOQ level (#b); and the BPB, BADGE•2H_2_O, BPF, BPZ, and BADGE•H_2_O•HCl were selectively detected in the samples (#c). 

The highest concentration of BPA among all analytes was found in sample No. 19 (3.63 ng/mL), followed by samples No. 18 (3.11 ng/mL), No. 17 (2.89 ng/mL), and No. 16 (2.44 ng/mL). Sample No. 6 contained the widest variety, with eight bisphenols detected: BPA, BPE, BADGE, BPAP, BADGE•2H_2_O, BPF, BPZ, and BADGE•H_2_O•HCl. Seven bisphenols were detected in samples No. 7, 17, 18, and 19. Six bisphenols, including BPA, BPE, BADGE, BPAF, BPZ, and BADGE•H_2_O•HCl were found in both pericardial fluid samples (nos. 1 and 2). Six bisphenols were detected in samples No. 5, 11, 12, and 16. Five bisphenols were detected in samples No. 3, 13, and 15. Samples No. 8, 9, and 14 contained four different analytes. BADGE•2H_2_O was detected only in samples No. 3, 6, 7, and 11. BPB and BADGE•2H_2_O were determined only in two samples (No. 7 and 11).

**Figure 6:**
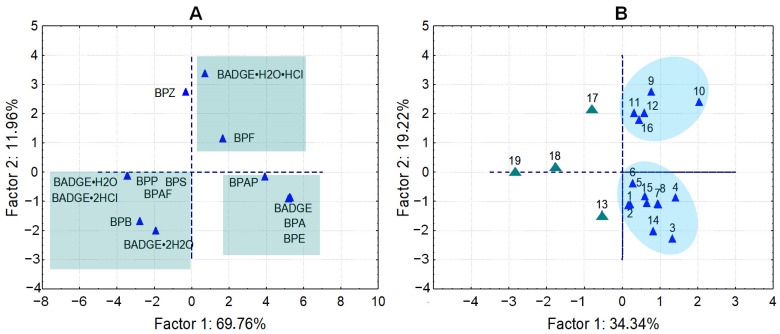
PCA score plots for bisphenols concentration (**A**) in pericardial fluids determined by LC–ESI–QqQ and patients (**B**) with coronary artery diseases.

The PCA components (Figure 6A) accounted for approximately 69% of the total variance in bisphenol distribution. The clustering of bisphenols shows a clear distinction and reflects a similar concentration distribution. Regarding the PCA (Figure 6B), two main groups of patients were observed. Samples No. 13, 17, 18, and 19 were arbitrarily plotted in the plot, highlighting a difference between these samples and the others. 

### 2.4. Discussion of the Clinical Data

#### 2.4.1. Description of Patients Included in the Present Studies with Eligibility Criteria

The study group consisted of 19 Polish patients from the Department of Cardiac Surgery: 7 women (aged between 66 and 76 years) and 11 men (aged between 47 and 76 years), all in a high-risk group for sudden death due to coronary artery disease. The patients were qualified for immediate coronary artery bypass surgery.

The primary indications for coronary artery bypass surgery were typically fixed narrowing(s) of the coronary vessels due to atheroma, partial blockage of the coronary arteries by thrombus, and the risk of their complete closure.

The inclusion criteria for coronary artery bypass surgery included the following: fixed narrowing(s) of the coronary vessels due to atheroma and/or confirmation of coronary artery or arteries partially blocked by thrombus. 

#### 2.4.2. Clinical Issues Related to the Occurrence of Bisphenols in Pericardial Fluid Samples

##### Introduction to Clinical Issues

Typical cases of disease in patients include cardiovascular (CV) system disease, such as angina, hypertension, myocardial infarction, and coronary and peripheral arterial disease.

Myocardial infarction occurs when a thrombus blocks a coronary artery. This can be fatal and is a common cause of death, often resulting from mechanical failure of the ventricle or from dysrhythmia. Cardiac myocytes rely on aerobic metabolism, and if the supply of oxygen falls below a critical threshold, a series of events leading to cell death begins. This process is clinically detected by an elevation of circulating troponin (a biochemical marker of myocardial injury) as well as by increased levels of cardiac enzymes (e.g., the cardiac isoform of creatinine kinase) and changes in the surface ECG. 

Preventing irreversible ischaemic damage following an episode of coronary thrombosis is critical. Opening the occluded artery as quickly as possible is essential. When logistically feasible, angioplasty (performed using a catheter with an inflatable balloon near its tip, with the administration of a glycoprotein IIb/IIIa antagonist to prevent reocclusion) is somewhat more effective than thrombolytic drugs, though the latter remains an effective alternative when angioplasty is unavailable.

Angina occurs when the oxygen supply to the myocardium is insufficient to meet its needs. The pain typically has a characteristic distribution in the chest, arm, and neck, and is brought on by exertion, cold, or emotional stress. 

Three kinds of angina are recognized clinically: stable, unstable, and variant.

Below is an attempt to link the results of bisphenol analysis with some of the most common cardiovascular disorders. 

##### Study of Data of Patients with Coronary Artery Diseases

**Figure 7:**
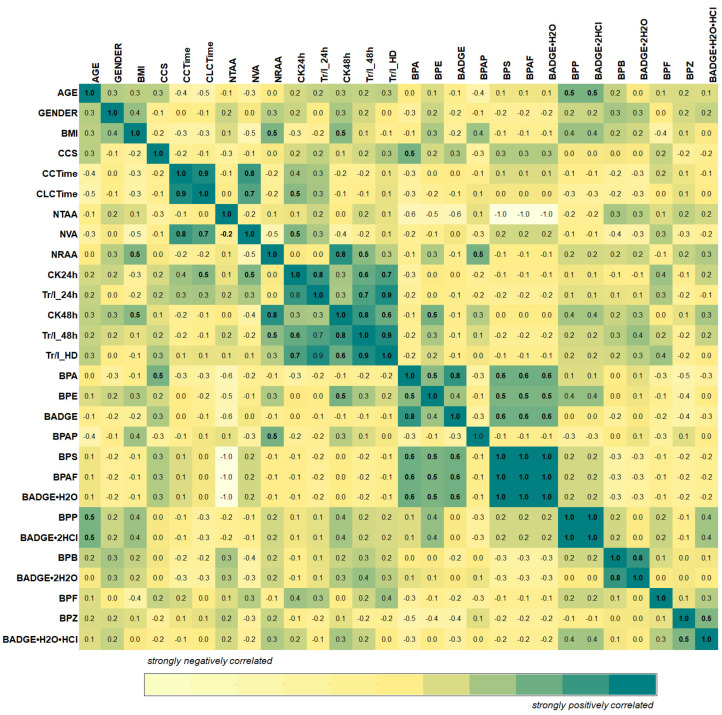
Heat map showing pairwise correlation matrix of the input variables (clinical data of 19 patients with coronary artery diseases) and quantified bisphenols. CCS/central cord syndrome; CCTime/circulation time (min); CLCTime/clamp cap time (min); NTAA/no of thoracic artery anastomoses; NVA/no of venous anastomoses; NRAA/no of radial artery anastomoses; CK24h/creatinine kinase (U/L), 24 h after the surgency; CK48h/creatinine kinase (U/L), 48 h after the surgency; Tr/I_24h/Troponin I (ng/L), 24 h after the surgency; Tr/I_48h/Troponin I (ng/L), 48 h after the surgency; Tr/I_HD/Troponin I (ng/L), Hospital discharge data.

The correlation matrix provides a visualization of the non-parametric dataset with various measurement units (Figure 7). As illustrated in Figure 7, all data were included in the analysis, as an in-depth and detailed examination of individual cases is of significant interest to patients with coronary artery disease undergoing coronary artery bypass surgery.

When assessing the first two factors, the age of the patients is important, while the gender of the patients from whom the pericardial fluid was collected is irrelevant. The correlation map revealed an additional similarity (r = 0.5) between age and bisphenols such as BPP and BADGE•2HCl. 

As shown in Figure 7, an examination of the relationships between the selected variables revealed a strongly positive correlation in clinical data between creatinine kinase (CK24h; CK48h) and troponin I (Tr/I_24h; Tr/I_48h) levels measured at 24 h and 48 h post-surgery. Additionally, strong positive correlations (r = 0.7 up to 0.90) were observed between troponin I levels at hospital discharge (Tr/I_HD) and CK24h, CK48h, and Tr/I_48h levels. The results align with observations in cardiac surgery, where higher postoperative troponin values typically correspond to higher CK-MB values.

Furthermore, creatinine kinase and troponin I values measured at 48 h post-surgery were correlated to BMI. A correlation was also found between the concentration of BPAP and the number (No.) of radial artery anastomoses (NRAA) (r ≥ 0.5).

A strong positive correlation was also observed between the number of venous anastomoses (NVA), circulation time (CCTime), and clamp cap time (CLCTime) (r ≥ 0.7). 

**Figure 8:**
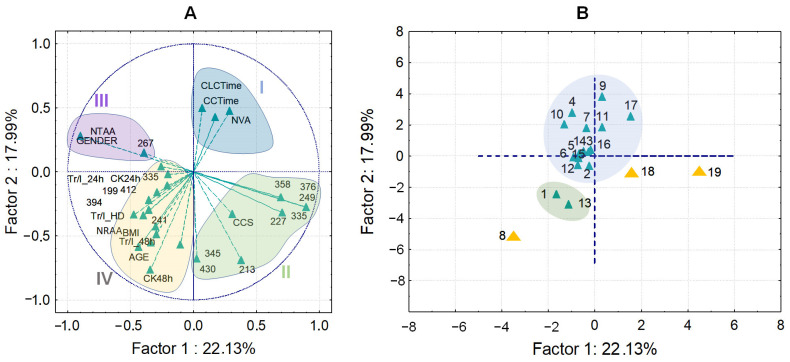
PCA score plots for clinical data and quantified bisphenols referred to 19 patients (**A**) and the scatter plot for patients (**B**) with coronary artery diseases. The numbers plotted in (**A**) represent the *m*/*z* of bisphenols while the numbers plotted in (B) represent the patients name. 227—BPA, 249—BPS, 241—BPB, 199—BPF, 345—BPP, 213—BPE, 267—BPZ, 335—BPAP, BPAF—335, 358—BADGE, 376—BADGE•H_2_O, 394—BADGE•2H_2_O, 412—BADGE•H_2_O•HCl, and 430—BADGE•2HCl. CCS/central cord syndrome; CCTime/circulation time (min); CLCTime/clamp cap time (min); NTAA/No. of thoracic artery anastomoses; NVA/No. of venous anastomoses; NRAA/No. of radial artery anastomoses; CK24h/creatinine kinase (U/L), 24 h after the surgency; CK48h/creatinine kinase (U/L), 48 h after the surgency; Tr/I_24h/Troponin I (ng/L), 24 h after the surgency; Tr/I_48h/Troponin I (ng/L), 48 h after the surgency; Tr/I_HD/Troponin I (ng/L), Hospital discharge data.

Moreover, the heat map (Figure 7) revealed an additional similarity (r = 0.5) between central cord syndrome (CCS) and bisphenol A (BPA). On the other hand, a strong negative relationship was observed between CCS and bisphenols (group II, Figure 8). 

Furthermore, the heat map (Figure 7) highlights the similarities among certain bisphenols, including BPA, BPE, BADGE, BPS, BPAF, BADGE•H_2_O, and BPB with correlation values of r ≥ 0.5. 

The analysis of the relationships between the clinical markers, patient characteristics, and bisphenols in the context of cardiac surgery provides valuable insights into postoperative recovery and potential environmental influences on health. The study highlights significant correlations among several biochemical and clinical parameters, offering a nuanced understanding of how variables such as age, body mass index (BMI), and bisphenol exposure might interact with key markers of cardiac injury. 

One of the notable findings from this study is the positive correlation between age and the concentration of certain bisphenols, including BPP and BADGE•2HCl (r = 0.5). This result suggests that as patients age, their exposure to or retention of bisphenols may increase, which could have implications for both cardiac health and recovery after surgery. Bisphenols are environmental pollutants commonly found in plastics and other materials, and evidence has been accumulating regarding their potential endocrine-disrupting properties. Given that aging often results in a variety of physiological changes, including alterations in metabolism and organ function, it is plausible that these compounds may have cumulative effects over time. Future research should explore whether age-related changes in bisphenol metabolism contribute to the increased incidence of cardiovascular diseases in older populations.

In contrast to age, gender did not show a significant correlation with any of the variables under study. This finding is consistent with previous research suggesting that gender, while a significant factor in some health outcomes, may not always have a direct relationship with the biomarkers analyzed in this study, particularly in the context of cardiac surgery recovery. The absence of gender as a factor in this analysis emphasizes the need for more individualized approaches in clinical practice, focusing on other patient-specific factors, such as age and BMI, which appear to play more prominent roles in postoperative recovery.

A central theme in this analysis is the strong positive correlation between troponin I levels and creatinine kinase (CK) values, both at 24 and 48 h post-surgery. Specifically, correlations between CK24h and Tr/I_24h, as well as between CK48h and Tr/I_48h, highlight the expected relationship between these biomarkers in the context of myocardial injury. Elevated troponin I and CK-MB levels are well-established markers of cardiac muscle damage, and the strong correlation between them supports their role in reflecting the extent of injury following cardiac surgery. Additionally, the correlations between troponin I and CK levels at hospital discharge (Tr/I_HD, CK24h, CK48h, Tr/I_48h) further underline their clinical relevance in postoperative monitoring. The interplay between these markers suggests that combining troponin I and CK measurements can provide a comprehensive understanding of cardiac recovery and injury, which could potentially improve patient outcomes by enabling more precise monitoring and intervention.

Another interesting observation is the correlation between BMI and postoperative levels of CK and troponin I, specifically at 48 h post-surgery. Obesity is a known risk factor for cardiovascular disease and is associated with worsened outcomes in cardiac surgery patients. The observed correlations imply that higher BMI may be linked to greater myocardial injury or a slower recovery process, as evidenced by the elevated levels of these markers. This underscores the importance of addressing BMI as part of preoperative risk stratification and postoperative care. Interventions aimed at managing BMI could potentially improve recovery trajectories in overweight or obese patients, although further investigation is needed to fully understand the mechanisms behind this relationship.

The study also found correlations between bisphenols and surgical parameters, such as the number of radial artery anastomoses (r ≥ 0.5 for BPAP) and the number of venous anastomoses, circulation time, and clamp cap time (r ≥ 0.7). These findings suggest that bisphenols may influence the surgical process or recovery in ways that have yet to be fully understood. Bisphenols, particularly BPA and its analogs, are known to have endocrine-disrupting effects that could impact vascular function and response to surgery. The correlation between BPAP and the number of radial artery anastomoses is of particular interest, as it may indicate a potential interaction between bisphenol exposure and vascular healing or remodeling. Given the increasing prevalence of bisphenol exposure in modern environments, it is crucial that future studies investigate how these compounds may interact with surgical outcomes, particularly in patients undergoing complex procedures like coronary artery bypass grafting.

One of the more intriguing aspects of this analysis is the relationship between central cord syndrome (CCS) and bisphenols. The positive correlation between CCS and bisphenol A (BPA) (r = 0.5) suggests that exposure to BPA may play a role in the development or exacerbation of neurological complications following cardiac surgery. However, the strong negative relationship between CCS and other bisphenols (group II) warrants further investigation. It is possible that different bisphenols have varying effects on neurological outcomes, with some compounds potentially offering protective effects while others may exacerbate damage. The complexities of these interactions highlight the need for further research to clarify the mechanisms behind bisphenol-induced neurological effects, especially in the context of patients undergoing major surgeries.

Finally, the analysis reveals strong positive correlations between various bisphenols, including BPA, BPE, BADGE, BPS, BPAF, BADGE•H_2_O, and BPB, with correlation coefficients ranging from r ≥ 0.5. These findings suggest that despite structural differences, many bisphenols share similar biochemical properties or mechanisms of action. This could have significant implications for regulatory standards and public health policies, as exposure to a range of bisphenols could collectively contribute to adverse health outcomes. Further research is needed to explore the cumulative effects of these chemicals and to assess the potential risks associated with long-term exposure.

This study underscores the complex relationships between age, bisphenol exposure, clinical markers, and surgical outcomes in cardiac surgery patients. The correlations identified in this analysis highlight the importance of considering environmental factors, such as bisphenol exposure, in the context of clinical recovery and cardiovascular health. The findings suggest potential pathways through which bisphenols may influence postoperative recovery, particularly in relation to myocardial injury and neurological complications. Future studies should aim to further elucidate the mechanisms underlying these associations and explore strategies to mitigate the effects of bisphenol exposure on patient health. Additionally, personalized care strategies that take into account factors such as age, BMI, and environmental exposures may be essential for improving patient outcomes following cardiac surgery.

The PCA components (Figure 8A) visualize four groups of the input variables. Group I indicates a strong positive relationship between the number of radial artery anastomoses, circulation time, and clamp cap time, highlighting their interrelatedness. In contrast, a strong negative relationship was observed among the variables in groups II and III. Group IV shows associations between clinical data and quantified bisphenols, providing insights into diagnostic interpretations for patients with coronary artery disease undergoing coronary artery bypass surgery.

Regarding the PCA (Figure 8B), patients were divided into three main, observable groups. Samples No. 8, 18, and 19 were arbitrarily positioned in the plot, suggesting differences from other patients. Patient No. 8, in particular, is notable for having the highest levels of creatinine kinase as well as high levels of troponin I 48 h post-surgery. This patient is further distinguished from other patients by the presence of radial artery anastomoses (NRAA) and the absence of venous anastomoses (NVA). 

Similarly, for patients No. 1 and No. 13, elevated creatinine kinase and high troponin I levels 48 h post-surgery were recorded (Figure 4). Conversely, a significant decrease in troponin I levels was observed at the time of hospital discharge (Figure 4). No influence of gender on the clinical data or bisphenol presence was detected.

1. Studies examining cardiovascular system diseases or hypertension

In this study, an attempt was made to link bisphenol frequency and concentrations in pericardial fluid collected during coronary artery bypass surgery with common causes of cardiovascular (CV) system disease. Both endogenous and predominantly exogenous factors may affect bisphenol residue levels in the pericardial fluid. The highest concentrations of BPA were detected in all samples from the 19 patients. Mild correlations were observed between bisphenol levels, their frequencies in pericardial fluid samples, and certain clinical data. 

After interpreting the results, the findings indicate that BPA was present in samples from patients who were older than the average age within the examined group. The highest concentrations of BPA were found in almost all samples collected from older or the oldest patients (more than 69 years of age), especially, No. 19 (3.63 ng/mL) and No. 18 (3.11 ng/mL), as well as No. 16 (2.44 ng/mL), No. 12 (2.04 ng/mL), No. 15 (1.83 ng/mL), and No. 13 (1.97 ng/mL). Additionally, high BPA levels were quantified in the pericardial fluid samples from six patients (No. 1, 3, 12, 16, 18, and 19) with the highest value of central cord syndrome (CCS equals 4). Furthermore, BPF, BPE, BPZ, BPAP, and BADGE•H_2_O•HCl were frequently quantified in samples collected from these patients with a CCS score of 4. The levels of other bisphenols (BPF, BPE, BPZ, BPAP, and BADGE•H_2_O•HCl) were quantified in samples collected from these patients with a CCS score of 4.

2. Cardiac troponin I concentration

Troponins are proteins that regulate the strength and speed of contraction in striated muscles, specifically in skeletal and cardiac muscle. Under normal conditions, cardiac troponins are absent from the bloodstream of healthy individuals. These proteins only enter the bloodstream when cardiac muscle cells are damaged, as occurs during a heart attack and other conditions leading to necrosis of heart muscle tissue. Due to its high sensitivity and specificity, measuring cardiac troponin concentration is currently the most critical laboratory test for diagnosing suspected heart attacks. 

There are three types of troponins: C, T, and I. All of these are responsible for regulating muscle contraction. Moreover, two specific forms are distinguished, the so-called cardiac troponins: (i) cardiac troponin T, and (ii) cardiac troponin I. For proper muscle contraction and relaxation, the presence of three types of troponins is necessary: troponin C, T, and I.

When muscle cells are damaged, their contents ‘spill out’ and troponins are released into the blood. This phenomenon is observed, among others, during a myocardial infarction, where troponin levels in the blood rise significantly (the greater the larger area of the muscle affected by the infarction) within as little as 3–4 h after the occurrence of the infarction. Troponin levels typically return to baseline values after approximately 10–14 days.

Testing the concentration of cardiac troponins is always recommended when symptoms occur that may suggest a suspicion of a heart attack: sharp, stabbing pain in the chest that may radiate to the left arm, jaw, neck, or back, shortness of breath, palpitations, cold sweats, feelings of anxiety, fainting, dizziness, or loss of consciousness. Testing troponin concentration is also used as a prognostic marker in patients with acute coronary syndrome—a disease that can lead to a heart attack—as well as in patients with chronic renal failure or post-heart transplantation).

As shown in Figure 4, when comparing cardiac troponin I concentrations of all patients on the day of surgery, in the first 24 h (to 24 h after surgery), and on the day of hospital discharge, a significant decrease in troponin I concentration in the blood was observed. As shown in Figure 4, when comparing concentrations of troponin I on the day of surgery (Tr/I_24h) and on the day of hospital discharge (Tr/l_HD), the concentration of troponin I decreased significantly: five-fold (No. 8); from seven-fold to nine-fold (No. 1, 2); ten-fold to fourteen-fold (No. 4, 5, 12, 13), twenty-one-fold (No. 19), thirty-five to thirty-nine-fold (No. 10, 11), forty-five to fifty-three-fold (No. 9, 16, 17), ninety-fold (No. 15), over one hundred-fold (No. 7, 14, 18), and over five hundred-fold (No. 3, 6).

A reduction in the concentration of cardiac troponin I in the blood of all patients discharged from the hospital (Department of Cardiac Surgery) indicates their complete recovery.

3. Studies examining overweight and obesity

This publication attempts to link the frequency and concentrations of bisphenols in pericardial fluid collected during coronary artery bypass surgery, and to explore the correlation between the frequency and concentrations of bisphenols with overweight and obesity. As shown in Figure 7, creatinine kinase and troponin I values determined at 48 h post-surgery are correlated to BMI. 

Bisphenols such as BPA, BPF, BPE, and BADGE were detected in pericardial fluid samples collected from a woman with the highest BMI value (>40). The concentrations of bisphenols were quantified in amniotic fluid samples collected from seven patients with BMI values greater than 30. These included: No. 5: BPA, BPF, BPE, BPAP, BADGE, and BADGE•H_2_O•HCl; No. 6: BPA, BPF, BPE, BPZ, BPAP, BADGE, BADGE•2H_2_O and BADGE•H_2_O•HCl; No. 10: BPA, BPE, and BADGE; No. 13: BPA, BPF, BPE, BPAP, and BADGE; No. 14: BPA, BPE, BPAP, and BADGE; No. 16: BPA, BPF, BPE, BPAP, BADGE, and BADGE•H_2_O•HCl; and No. 18: BPA, BPF, BPE, BPZ, BPAP, BADGE, and BADGE•H_2_O•HCl. In contrast, concentration residues of some of analytes (BPA, BPF, BPE, BPZ, BPAP, BADGE, and BADGE•H_2_O•HCl) were found in the sample collected from a man (No. 17) with the lowest BMI value (<24). 

Of course, the present study has some limitations. First, the study group consisted of a relatively small number of women and men with coronary artery disease who were indicated for coronary artery bypass surgery. Second, the sample size was limited to a relatively small number of patients. Therefore, further studies with a larger sample size are needed to confirm the observed relationships. The preliminary results of this study suggest that the associations between the concentration of xenobiotics from the bisphenols group in pericardial fluid samples warrant further investigation in large-scale human biomonitoring studies.

4. Additional information about the study cohort group

Stable angina pectoris was diagnosed in six patients (No. 1–4, 9, 13), and unstable angina pectoris was observed in three (No. 8, 12, 14). One patient had two myocardial infarctions (2) and six patients had one myocardial infarction (No. 5–7, 10, 12, 15) prior to the planned procedure.

One patient (No. 9) suffered a perioperative myocardial infarction during the procedure. 

The level of glycated hemolobin (HbA1c) was also monitored before the planned surgery, which is crucial for patients with suspected or diagnosed diabetes (type 1 or 2). The normal level of glycated hemolobin HbA1c in patients with type 1 diabetes or short-term type 2 diabetes is 6.5% (equal to or less than 48 mmol/mol). In children and adolescents, the normal level of glycated hemolobin HbA1c is also 6.5% (equal to or less than 48 mmol/mol). In elderly individuals with complications of a macrovascular nature (previous heart attack and/or stroke) and/or numerous comorbidities, the normal concentration of glycated hemoglobin HbA1c is equal to or less than 8% (equal to or less than 64 mmol/mol).

Other diabetic patients and people over 65 years of age who are expected to live more than 10 years typically have a normal concentration of glycated hemoglobin HbA1c equal to or less than 7% (equal to or less than 53 mmol/mol).

The concentration of glycated hemoglobin HbA1c in the study group (in patients) was as follows: 5.30% (No. 12), 5.40% (No. 3 and 11), 5.60% (No. 14), 5.90% (No. 6), 6.00% (No. 2), 7.20% (No. 7), and 8.80% (No. 10).

## 3. Materials and Methods

### 3.1. Bisphenols Standards and Chemical Reagents

All bisphenol standards: bisphenol A (CAS: 80-05-7), bisphenol F (CAS: 620-92-8), bisphenol E (2081-08-5), bisphenol P (2167-51-3), bisphenol A diglycidyl ether (BADGE; CAS 1675-54-3), BADGE·2H_2_O (CAS: 5581-32-8), BADGE·2HCl (CAS: 4809-35-2) with purity ≥ 98% were purchased from Sigma Aldrich (Bellefonte, MO, USA).

Solvents used during the procedure, such as methanol (MeOH), acetonitrile (ACN), dichloromethane (CH_2_Cl_2_) and acetone with LC–MS purity were obtained from Sigma Aldrich (St. Louis, MO, USA). Formic acid (HCOOH) used during HPLC–FLD studies was also obtained from Sigma Aldrich (St. Louis, MO, USA). Deionized water for the HPLC–FLD experiments was obtained in our laboratory using the Hydrolab System, (Gdańsk, Poland). For the LC–MS/MS analysis, water with LC–MS purity was obtained from Merck (Darmstadt, Germany).

The stock solutions of each analyte (5000 ng/mL) were prepared in MeOH in a glass flask and stored in the freezer (−23 °C). The appropriate concentrations were achieved by diluting the primary solution with MeOH.

### 3.2. Sample Collection and Storage

After sternotomy, the first procedure during coronary artery bypass surgery is to collect a sample of fluid from the pericardial sac. Samples were stored in a freezer (−23 °C) and thawed before use in the procedure.

### 3.3. Sample Preparation Procedure

**Figure 9:**
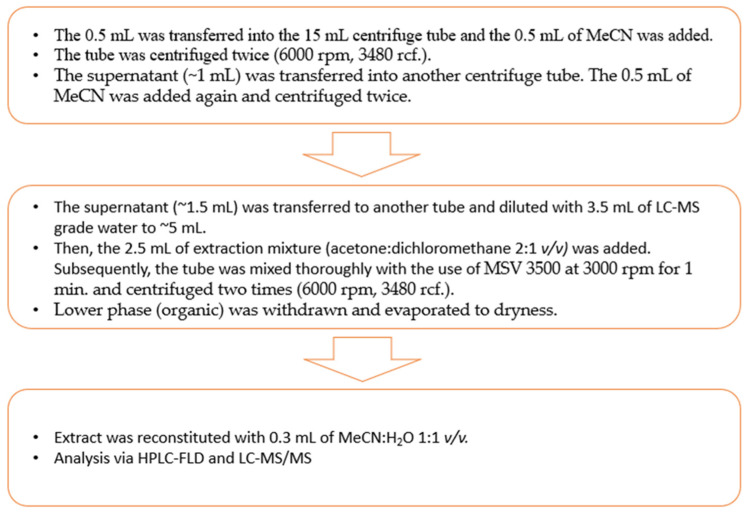
The flowchart of DLLME procedure.

Sample preparation was based on the dispersive liquid–liquid microextraction (DLLME) technique. The flowchart of the final procedure is presented in Figure 9. This procedure was based on previously published work [18]. During the optimization process, an additional deproteinization step with 0.5 mL of MeCN was added, and the volume of the extraction mixture was increased from 2.0 mL to 2.5 mL.

#### Extraction Recovery Studies, Accuracy, and Precision

Mean recoveries were evaluated at three different concentration levels: 10 ng/mL, 20 ng/mL, and 30 ng/mL of the sample. Mean recovery values were obtained from six replicates for every spiking levels. Accuracy in all cases was expressed as percentage recovery of the analyte and calculated using the formula showing in Supplementary Materials (Supplementary Materials Equation (S1)). Relative standard deviation was calculated using the formula showing in Supplementary Materials also (Supplementary Materials Equation (S2)).

Intra-day accuracy and precision was studied by analyzing samples in six replicases. The experiment in three following days. The results in showing in Tables S2 and S3.

### 3.4. HPLC–FLD: Instrumental Analysis and Chromatographic Conditions

The chromatographic equipment was the same and the gradient settings were set based on the previously published method [20].

The chromatographic equipment consisted of a quaternary pump (Agilent 1200), an autosampler with a thermostat (Agilent 1260 Infinity II Vialsampler), a column thermostat (Agilent 1200), and a fluorescence detector (Agilent 1260).

Separation was performed on a Scherzo SM-C18 (150 mm × 4.6 mm) column with a 3 µm particle size (Agilent Technologies, Wilmington, DE, USA), and thermostated at 22 °C. The mobile phase consisted of 50 mM formic acid (HCOOH) in water (component A) and 50 mM HCOOH in ACN. The gradient elution: 0–15 min, component B was increased from 40% to 75%; from 15 to 15.5 min., component B was increased from 75% to 85%; and from 15.5 to 20 min., an isocratic elution with 85% component B. The flow rate was 0.45 mL/min, and the samples were thermostated in the autosampler at 8 °C.

After each sample injection, the column was washed with 100% component B at a flow rate of 1.0 mL/min, followed by column conditioning with the initial isocratic elution composition (40% component B) for 15 min.

#### Selectivity

The selectivity evaluation was based on comparing the chromatograms from the averaged human pericardial fluid sample blank, the spiked human pericardial fluid sample before the extracting procedure, and the spiked human pericardial fluid after the procedure. Identification of bisphenols was based on comparing the retention times in the sample and in the mixture of bisphenols standards. Analysis was performed at 4 different excitation wavelengths: 225 nm, 230 nm, 235 nm, and 240 nm, with at 4 different the emission wavelengths: 300 nm, 305 nm, 310 nm, and 315 nm. The excitation wavelength of 240 nm and an emission wavelength of 300 nm were found as the most optimal variation in operation of the fluorescence detector. 

### 3.5. LC–MS/MS Analysis

LC–MS/MS analysis was performed using a triple quadrupole mass spectrometer (8050 Shimadzu, Kyoto, Japan) equipped with LabSolutions version 5.8 software for data collection and instrumental control. Electrospray ionization (ESI-/ESI+) was applied in both negative and positive ion modes. The ESI–MS/MS spectrometer was coupled with an UHPLC system (LC-30AD binary solvent delivery system, SIL-30AC autosampler, and CTO-20AC thermostat) (Kyoto, Japan). Prior to analysis, the mass spectrometer was calibrated using the manufacturer’s calibration solution. Data were acquired in full scan profile mode (m/z 50–600). In positive mode, [M+NH_4_]^+^ was selected as the precursor ion, whereas in negative mode, the precursor ion was [M–H]^−^. All BADGEs tend to form adducts in positive ESI mode; namely, ammonium adduct ions were obtained. In negative ESI mode, MeOH–water with no additives was used as the mobile phase to prevent ion suppression.

The ions were detected using multiple reaction monitoring (MRM) mode. Positive and negative ion modes were both employed for compound analysis. The optimization of different MS parameters on the selectivity and MS response (MRM peak areas) for the studied compounds was carried out without a chromatographic column (Table 1). 

To select the MS/MS parameters, standard solutions of the studied bisphenols at a concentration of 500 ng/mL were infused into the mass spectrometer using a Harvard syringe pump at a flow rate of 10 μL/min. The optimal parameters were as follows: interface temperature 300 °C, DL temperature 250 °C, heat block temperature 400 °C, nebulizing gas flow 2 L/min, drying gas flow 10 L/min, heating gas flow 10 L/min, interface voltage 3.5–4.5 kV, interface current 0.7 μA, and drying gas temperature 290–350 °C. Nitrogen was used as the collision gas, and the energy was set at 20–35 eV.

The analysis was performed using a Kinetex C18 analytical column (100 mm × 2.1 mm, 1.7 µm) with column temperature set at 30 °C. The mobile phase consisted of water (mobile phase A) and methanol (mobile phase B) (for BPS, BPF, BPE, BPA, BPAF, BPP, BPZ, BPB, BPAP) and 40 mM ammonium formate in water and methanol (for BADGE•2H_2_O, BADGE•H_2_O, BADGE•H_2_O•HCl, BADGE•2HCl, BADGE). The gradient program for the mobile phase was as follows: 0–1 min, 25% B; 1–5 min, linear to 95% B; then, the system returned to the initial conditions at a flow rate of 0.4 mL/min, with a stop time of 7 min and a post-time of 3 min. The injection volume was set to 1 µL, and the autosampler’s temperature was set at 4 °C.

#### LC–MS/MS: Method Validation

Validation parameters, including calibration data (calibration equations, linearity presented as a correlation coefficient (R^2^) of the calibration curves, limits of detection (LOD), limits of quantification (LOQ), and precision (RSD) are presented in Table 2.

The calibration range for the analyzed bisphenols was 0.04–100 ng/mL, meeting the requirements for the analytical method validation and ensuring accurate determination of linearity within this range. The LOD and LOQ values were calculated using the equations 3.3× (SD/S) and 10× (SD/S), respectively, where SD is the standard deviation of the response (peak area) and S is the slope of the calibration curve. Analyte identification was based on retention times. Each HPLC analysis was performed in triplicate. BPA-d_3_ (bisphenol A-d_3_), an isotopically labeled analog of bisphenol A (BPA), where three hydrogen atoms are replaced by their heavier isotope, deuterium (^2^H, denoted as “d”), was used as the internal standard.

### 3.6. Methods of Statistical Analysis

Statistical analysis was performed using Statistica v. 7 and Microsoft Excel 2010 software. The clustering method was employed to evaluate dissimilarities between quantified bisphenols in pericardial fluid samples collected from 19 patients. Data were clustered using the single linkage method, with a heat map generated through Excel’s conditional formatting. Relevant differences between variables were further assessed using principal components and classification analysis (PCA). The density plot was visualized using “plot var. pca factor coordinates 2d” and “plot case factor coordinates” models. Additionally, a correlation matrix was generated by Statistica v. 7 and zero-order correlations between all variables were considered. Pearson’s correlation coefficients (r) were interpreted within a range of −1 to 1, with statistical significance set at *p* < 0.05. 

## 4. Conclusions

A sensitive, accurate, and precise methodology was established for the determination of selected bisphenols in pericardial fluid samples at trace levels. To the best of our knowledge, this method is the first to combine the advantages of DLLME as an extraction technique with HPLC–FLD and LC–ESI–QqQ for the identification and quantification of analytes in pericardial fluid collected from patients with coronary artery disease undergoing coronary artery bypass surgery. The presented DLLME-based sample preparation technique, coupled with HPLC–FLD and LC–QqQ analysis, provides a reliable analysis of bisphenols in human pericardial fluid. By reducing the use of harmful solvents for both humans and the environment, the method can be classified as ‘green’. Given its low matrix effect, high recovery values, and repeatability, the DLLME procedure has been successfully combined with LC–ESI–QqQ (triple quadrupole spectrometer) to analyze 14 bisphenols in pericardial fluids collected from 19 patients undergoing coronary artery bypass surgery. Residues of ten analytes (BPA, BPB, BPF, BPE, BPZ, BPAP, BADGE, BADGE·2H_2_O, BADGE·H_2_O·HCl, and BADGE·2HCl) were detected in pericardial fluid collected from 19 patients undergoing coronary artery bypass surgery. 

Statistical analysis revealed a strong positive correlation between the number of venous anastomoses (NVA), circulation time (CCTime), and clamp cap time (CLCTime). This correlation is expected, as an increase in the number of anastomoses will always correlate with the clamp time and circulation time, because if more anastomoses are performed during cardiac surgery, the time of procedure of cardiac surgery increases.

Creatinine kinase and troponin I values determined at 48 h post-surgery are correlated with BMI. A similarity between BPAP levels and the number of radial artery anastomoses (NRAA) (r ≥ 0.5) was also observed. The heat map revealed a correlation (r = 0.5) between central cord syndrome (CCS) and bisphenol A (BPA). 

In the conducted research, BPA-d_3_, a deuterated analog of BPA, was used as an internal standard. This standard compensates for sample preparation losses and detector variations, ensuring precise calibration and accurate quantification in mass spectrometry analysis. The isotopic mass difference between BPA and BPA-d_3_ allows for easy differentiation, enhancing the reliability of the results.

Four bisphenols such as BPA, BPF, BPE, and BADGE were detected in the pericardial fluid sample collected from a woman with the highest BMI value (>40). A 67-year-old woman was diagnosed with coronary artery disease (three-vessel disease). Other preoperative selected data of this patient were as follows: CCS equals 3, the concentration of glycated hemoglobin HbA1c equals 5.70%, CRP equals 17.682 mg/L. These improved after cardiac surgery (undergoing coronary artery bypass surgery). Troponin I concentration to 24 h was 5371 ng/L, at 48 h post-surgery was 12,203 ng/L and at discharge from the hospital after 8 days it was 1123 ng/L.

The concentrations of bisphenols (from three to eight analytes) were quantified in pericardial fluid samples collected from six patients with BMI values greater than 30 and one less than 30. After this study, we concluded that in pericardial fluid samples collected from seven patients includes six with BMI values greater than 30 quantified BPA, BPE, and BADGE in all seven samples, BPAP in six samples, BPF in five samples, BADGE•H_2_O•HCl in four samples, BPZ in two samples, and BADGE•2H_2_O in one sample. Eight bisphenols such as BPA, BPF, BPE, BPZ, BPAP, BADGE, BADGE•2H_2_O, and BADGE•H_2_O•HCl were detected in the pericardial fluid sample collected from a 75-year-old woman with diagnosed coronary artery disease (three-vessel disease), and with BMI value equals 35.06. Six bisphenols such as BPA, BPF, BPE, BPAP, BADGE, and BADGE•H_2_O•HCl were detected in the pericardial fluid sample collected from a 70-year-old woman with also diagnosed coronary artery disease (three-vessel disease), and with BMI value equals 37.25. Also six bisphenols such as BPA, BPF, BPE, BPAP, BADGE, and BADGE•H_2_O•HCl were detected in the pericardial fluid sample collected from an 80-year-old woman diagnosed with three-vessel disease, and with BMI value equals 31.25. Five bisphenols such as BPA, BPF, BPE, BPAP, and BADGE were detected in pericardial fluid sample collected from a 71-year-old woman with diagnosed coronary artery disease (three-vessel disease), and with a BMI value of 34.14. 

Fewer bisphenols (BPA, BPE, and BADGE) were detected in the pericardial fluid sample collected from a 62-year-old man diagnosed with three-vessel disease, and with a BMI value of 34.02. Also, fewer bisphenols (BPA, BPE, BPAP, and BADGE) were detected in the pericardial fluid sample collected from a 66-year-old man with diagnosed coronary artery disease (two-vessel disease), and with a BMI value of 30.51. In contrast, concentration residues of seven analytes (BPA, BPF, BPE, BPZ, BPAP, BADGE, and BADGE•H_2_O•HCl) were found in the sample collected from a patient with the lowest BMI value of 23.51.

To sum up, despite the fact that the group of patients was not very large, a correlation can be observed between the BMI values above 30, and the occurrence of bisphenols and their concentrations in pericardial fluid samples in women over 67 years of age with diagnosed coronary artery disease (three-vessel disease). In men with diagnosed coronary artery disease with three or two-vessel disease over 62 years of age, with BMI values above 30, bisphenols were also determined, but less were identified in the pericardial fluid samples (three or four analytes). 

Future studies should focus on identifying populations that may be at the greatest risk for bisphenol-related health effects, in order to validate or challenge these preliminary findings.

## Figures and Tables

**Table 1:** MS/MS conditions used for determination and identification of selected bisphenols in biological samples.

Compound	Parent Ion, *m*/*z*	Quantifier ion [*Q*1], *m*/*z* Qualifier Ion [*Q*3], *m*/*z*	Collision Energy (eV); *Q1*, *Q3*	Drying Gas Temperature (°C)	Capillary Voltage (V)
**[M−H]^−^**					
**BPS** *Bis(4-hydroxyphenyl)sulfone*	249	108 156	22, 35	320	3500
**BPF** *4,4′-Methylenediphenol*	199	93 105	27, 24	320	4000
**BPE** *4-[1-(4-hydroxyphenyl)ethyl]phenol*	213	198 119	29, 35	320	4000
**BPA** *2,2-Bis(4-hydroxyphenyl)* *propane*	227	211 133	29, 30	290	4500
**BPB** *4-[2-(4-hydroxyphenyl)butan-2-yl]phenol*	241	226 212	29, 30	290	4500
**BPP** *4-[2-[4-[2-(4-hydroxyphenyl)propan-2-yl]phenyl]propan-2-yl]phenol*	345	330 131	28, 29	320	4000
**BPZ** *4-[1-(4-hydroxyphenyl)cyclohexyl]phenol*	267	173 145	26, 33	320	3500
**BPAF** *4-[1,1,1,3,3,3-hexafluoro-2-(4-hydroxyphenyl)propan-2-yl]phenol*	335	265 245	31, 28	290	4500
**BPAP** *4-[1-(4-hydroxyphenyl)-1-phenylethyl]phenol*	335	289 265	33, 30	290	4500
**[M + NH_4_]^+^**					
**BADGE 2H_2_O** *2,2-Bis [4-(2,3-dihydroxypropoxy)pheny]* *propane* *Bisphenol A Bis(2,3-dihydroxypropyl) ether*	394	209 135	25, 29	320	3500
**BADGE H_2_O** *3-[4-[2-[4-(oxiran-2-ylmethoxy)phenyl]propan-2-yl]phenoxy]propane-1,2-diol*	376	209 135	29, 31	320	3500
**BADGE H_2_O HCl** *3-[4-[2-[4-(3-chloro-2-hydroxypropoxy)phenyl]propan-2-yl]phenoxy]propane-1,2-diol*	412	227 191	32, 27	350	4000
**BADGE 2HCl** *2,2-Bis [4-(3-chloro-2-hydroxypropoxy)phenyl]propane* *Bisphenol A Bis(3-chloro-2-hydroxypropyl) ether*	430	227 135	26, 29	350	3500
**BADGE** *2,2-Bis [4-(glycidyloxy)* *phenyl]propane* *Bisphenol A diglycidyl ether*	358	191 161	30, 24	320	4500

**Table 2:** Calibration data of detected components including: calibration equations, linearity coefficient (R2), LOD, LOQ, and precision (RSD).

Compound	Regression Equation	R^2^	RSD%	LOD (ng/mL)	LOQ (ng/mL)
BPS	y = 15,061x + 5799	0.9998	2.42	0.27	0.81
BPF	y = 5993x + 348	0.9995	2.34	0.21	0.63
BPE	y = 7495x + 1740	0.9995	1.03	0.04	0.12
BPA	y = 9721x + 603	0.9993	2.15	0.06	0.18
BPB	y = 3398x + 645	0.9995	2.14	0.19	0.57
BPP	y = 4957x + 405	0.9993	1.98	0.06	0.18
BPZ	y = 7129x + 508	0.9995	1.74	0.17	0.51
BPAF	y = 4730x + 435	0.9993	1.90	0.06	0.18
BPAP	y = 4809x + 236	0.9996	1.98	0.08	0.24
BADGE•2H_2_O	y = 5025x + 511	0.9996	1.11	0.37	1.11
BADGE•H_2_O	y = 11,663x + 149	0.9996	0.93	0.28	0.84
BADGE•H_2_O•HCl	y = 4787x + 289	0.9993	1.46	0.21	0.63
BADGE•2HCl	y = 3816x + 869	0.9998	1.86	0.15	0.45
BADGE	y = 5043x 308	0.9997	2.52	0.11	0.33

## Data Availability

Data are contained within the article and Supplementary Materials. Authors are willing to share the original data and materials if so requested.

## Supplementary Materials

The following supporting information can be downloaded at: https://www.mdpi.com/article/10.3390/molecules30010140/s1, Figure S1: Chromatogram of the matrix after procedure with application dispersive liquid–liquid microextraction (DLLME) method; Figure S2: QqQ-ESI-MS (top) and MS/MS (bottom) spectra of following bisphenols residues detected in pericardial fluid samples: (a) BPS (*m*/*z* = 249), (b) BPF (*m*/*z* = 199), (c) BPE (*m*/*z* = 213), (d) BPA (*m*/*z* = 227), (e) BPB (*m*/*z* = 241), (f) BPP (*m*/*z* = 345), (g) BPZ (*m*/*z* = 267), (h) BPAF (*m*/*z* = 335), (i) BPAP (*m*/*z* = 335), (j) BADGE•2H_2_O (*m*/*z* = 394), (k) BADGE•H_2_O (*m*/*z* = 376), (l) BADGE•H_2_O•HCl (*m*/*z* = 412), (m) BADGE•2HCl (*m*/*z* = 430), (n) BADGE (*m*/*z* = 358); Table S1: Analysis of selected bisphenols in pericardial fluids collected from 19 patients with coronary artery diseases and undergoing coronary artery bypass surgery with the use of LC–ESI–QqQ; Table S2: Validation of the HPLC–FLD method after DLLME method. Intra and inter-day accuracy (Recovery %) and precision (RSD%), and intra-laboratory reproducibility; Table S3: Validation of the HPLC–FLD method after DLLME procedure. Intra-day accuracy and precision for fortified level at 25 ng mL^−1^ (additional results). Extraction Recovery Studies, Accuracy, and Precision with Equations (S1) and (S2).
