# Supplementary material for: Potential Clinical Application of Analysis of Bisphenols in Pericardial Fluid from Patients with Coronary Artery Disease with the Use of Liquid Chromatography Combined with Fluorescence Detection and Triple Quadrupole Mass Spectrometry
Source: Molecules. 2025 Jan 1;30(1):140. doi: 10.3390/molecules30010140 (PMC11722154; doi:10.3390/molecules30010140)
Supplement: Supplementary file 1 [file molecules-30-00140-s001.zip › molecules-3348808-supplementary.pdf]

Supplementary Material to

molecules MDPI

Article

Potential Clinical Application of Analysis of Bisphenols in Pericardial Fluid from Patients with Coronary Artery Disease with the use of Liquid Chromatography Combined with Fluorescence Detection and Triple Quadrupole Mass Spectrometry

Tomasz Tuzimski[1*], Szymon Szubartowski[1,2], Janusz Stążka[3], Kamil Baczewski[3], Daria Janiszewska[4], Viorica Railean[5,6], Bogusław Buszewski[4,7], Małgorzata Szultka-Młyńska[4]

[1] Department of Physical Chemistry, Chair of Chemistry, Faculty of Pharmacy, Medical University of Lublin, Chodźki 4a, 20-093 Lublin, Poland; tomasz.tuzimski@umlub.pl https://orcid.org/0000-0003-3729-3196
[2] Doctoral School of Medical University of Lublin, Medical University of Lublin, Chodźki 6, 20-093 Lublin, Poland; szymon.szubartowski95@gmail.comhttps://orcid.org/0000-0003-0303-9624
[3] Department of Cardiac Surgery, Medical University of Lublin, Jaczewskiego 8 (USK Nr 4), 20-093 Lublin, Poland; janusz.stazka@umlub.plhttps://orcid.org/0000-0001-8106-8346 ; kamil.baczewski@umlub.pl https://orcid.org/0000-0002-2394-3699
[4] Department of Environmental Chemistry and Bioanalytics, Gagarina 7, Faculty of Chemistry, Nicolaus Copernicus University, 87-100 Torun, Poland; janiszewska_daria@doktorant.umk.pl https://orcid.org/0000-0002-5252-6886 ; mszultka@umk.plhttps://orcid.org/0000-0002-4499-0128 ; bbusz@chem.umk.pl https://orcid.org/0000-0002-5482-7500
[5] Department of Infectious, Invasive Diseases and Veterinary Administration, Institute of Veterinary Medicine, Nicolaus Copernicus University in Torun, Gagarina 7, 87-100 Toruń, Poland; viorica.railean@umk.pl https://orcid.org/0000-0001-9916-0095
[6] Centre for Modern Interdisciplinary Technologies, Nicolaus Copernicus University, Wilenska 4, 87-100 Torun, Poland; viorica.railean@umk.plhttps://orcid.org/0000-0001-9916-0095
[7] Professor Jan Czochralski Kuyavian-Pomeranian Scientific Technological Centre, Krasinskiego 4, 87-100 Torun, Poland; bbusz@chem.umk.pl
* Correspondence: e-mail tomasz.tuzimski@umlub.pl; Tel.: +48 814487213

Figure S1: Chromatogram of the matrix after procedure with application dispersive liquid–liquid microextraction (DLLME) method.

Figure S2. QqQ-ESI-MS (top) and MS/MS (bottom) spectra of following bisphenols residues detected in pericardial fluid samples: (**a**) BPS (*m*/*z* = 249), (**b**) BPF (*m*/*z* = 199), (**c**) BPE (*m*/*z* = 213), (**d**) BPA (*m*/*z* = 227), (**e**) BPB (*m*/*z* = 241), (**f**) BPP (*m*/*z* = 345), (**g**) BPZ (*m*/*z* = 267), (**h**) BPAF (*m*/*z* = 335), (**i**) BPAP (*m*/*z* = 335), (**j**) BADGE•$2H_2O$ (*m*/*z* = 394), (**k**) BADGE•H_2O (*m*/*z* = 376), (**l**) BADGE•H_2O•HCl (*m*/*z* = 412), (**m**) BADGE•2HCl (*m*/*z* = 430), (**n**) BADGE (*m*/*z* = 358).

Table S1. Analysis of selected bisphenols in pericardial fluids collected from 19 patients with coronary artery diseases and undergoing coronary artery bypass surgery with the use of LC-ESI-QqQ.

Table S2. Validation of the HPLC-FLD method after DLLME method. Intra and inter-day accuracy (Recovery %) and precision (RSD%), and intra-laboratory reproducibility.

Table S3. Validation of the HPLC-FLD method after DLLME procedure. Intra-day accuracy and precision for fortified level at 25 ng mL^{-1} (additionally results).

Extraction Recovery Studies, Accuracy and Precision with Eq. (S1) and Eq. (S2).

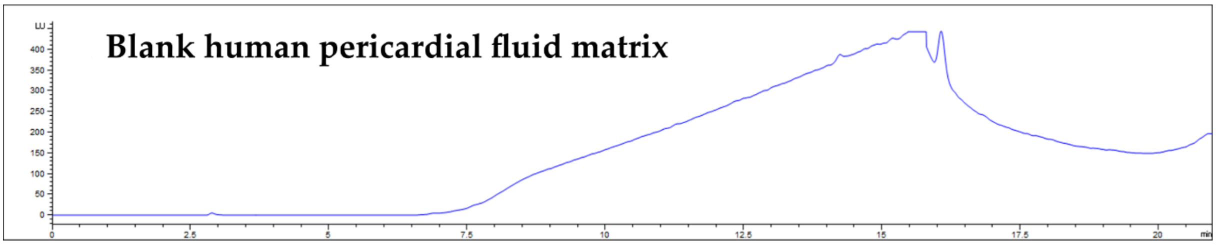

Figure S1: Chromatogram of the matrix after procedure with application dispersive liquid–liquid microextraction (DLLME) method.

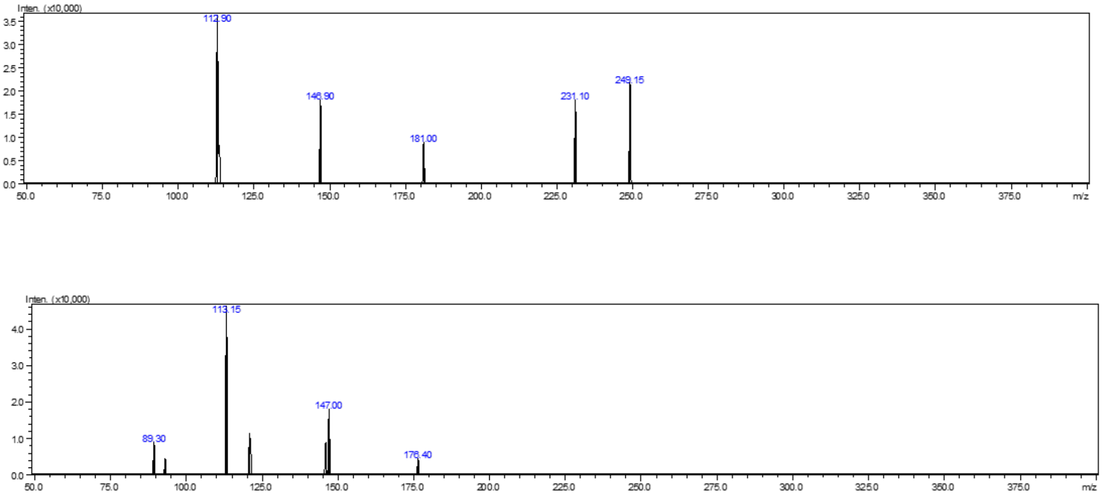

Figure S2a. QqQ-ESI-MS (top) and MS/MS (bottom) spectra of BPS (m/z = 249) detected in pericardial fluid samples.

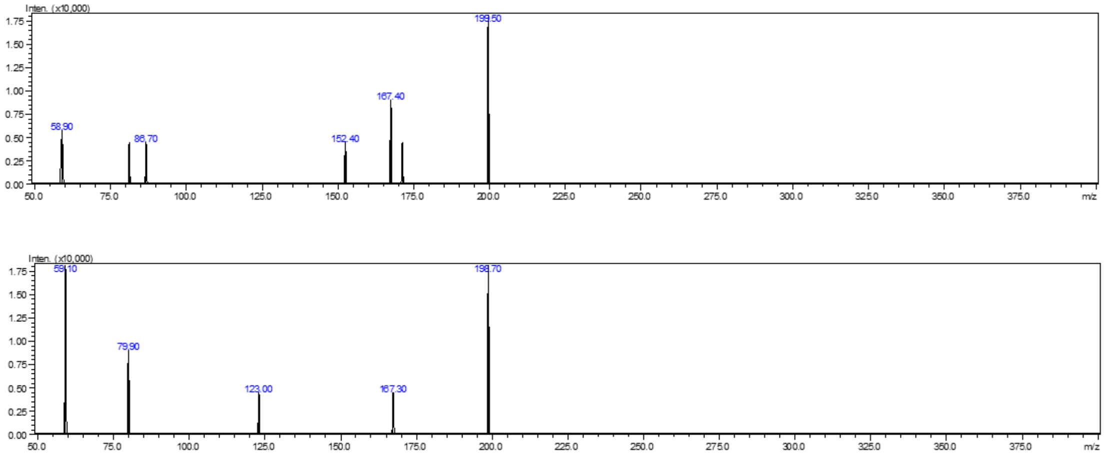

Figure S2b. QqQ-ESI-MS (top) and MS/MS (bottom) spectra of BPF (*m*/*z* = 199) detected in pericardial fluid samples.

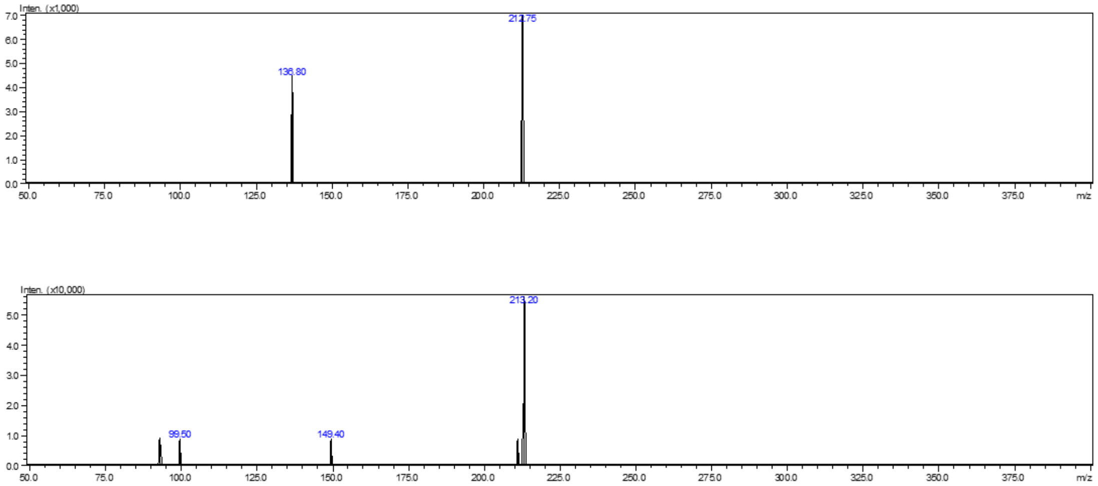

Figure S2c. QqQ-ESI-MS (top) and MS/MS (bottom) spectra of BPE (*m*/*z* = 213) detected in pericardial fluid samples.

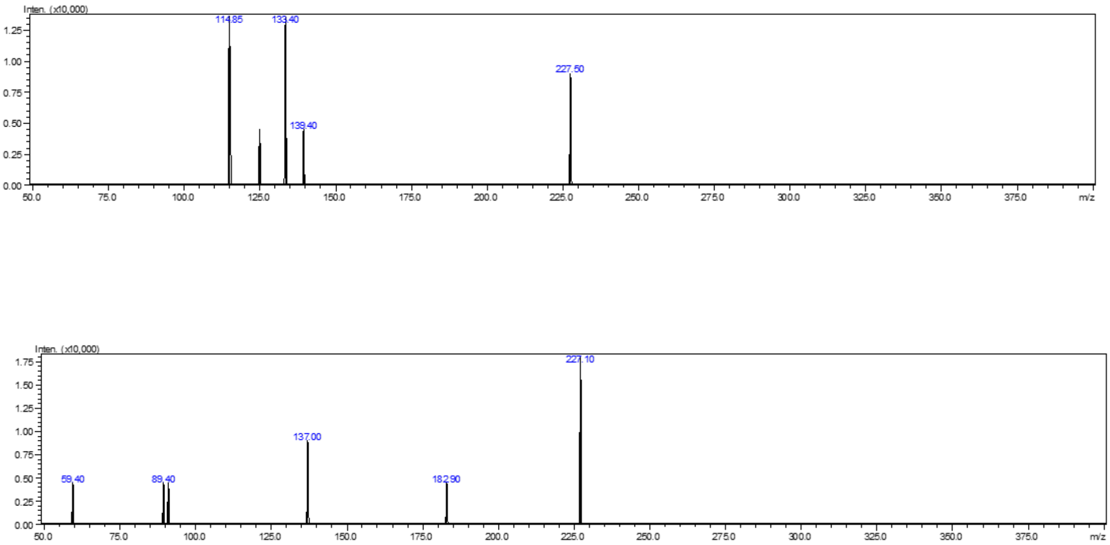

Figure S2d. QqQ-ESI-MS (top) and MS/MS (bottom) spectra of BPA (*m*/*z* = 227) detected in pericardial fluid samples.

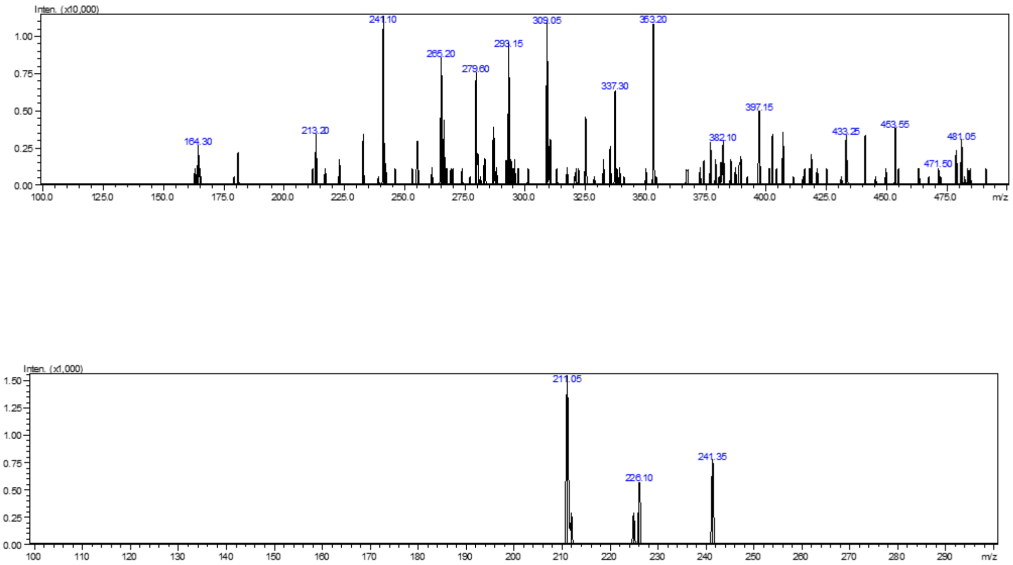

Figure S2e. QqQ-ESI-MS (top) and MS/MS (bottom) spectra of BPB (*m*/*z* = 241) detected in pericardial fluid samples.

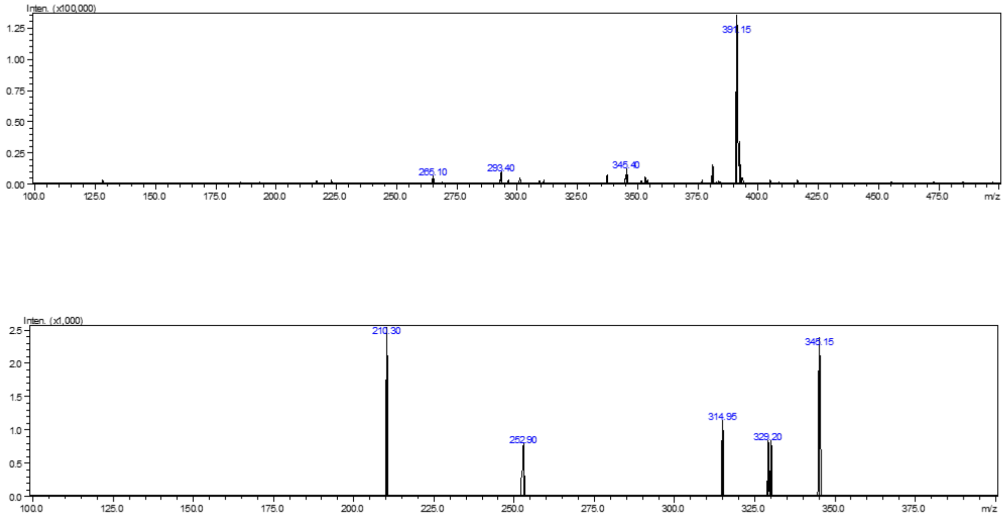

Figure S2f. QqQ-ESI-MS (top) and MS/MS (bottom) spectra of BPP (m/z = 345) detected in pericardial fluid samples.

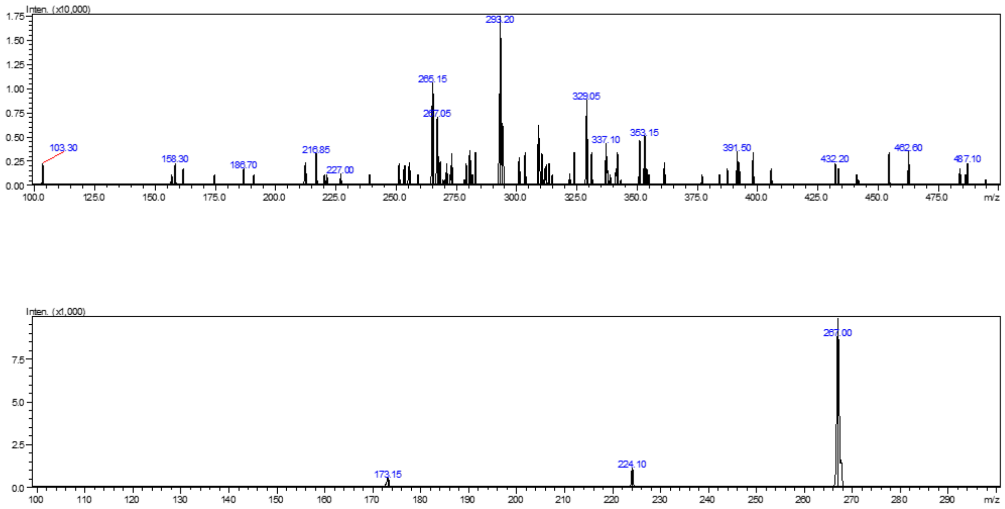

Figure S2g. QqQ-ESI-MS (top) and MS/MS (bottom) spectra of BPZ (*m*/*z* = 267) detected in pericardial fluid samples.

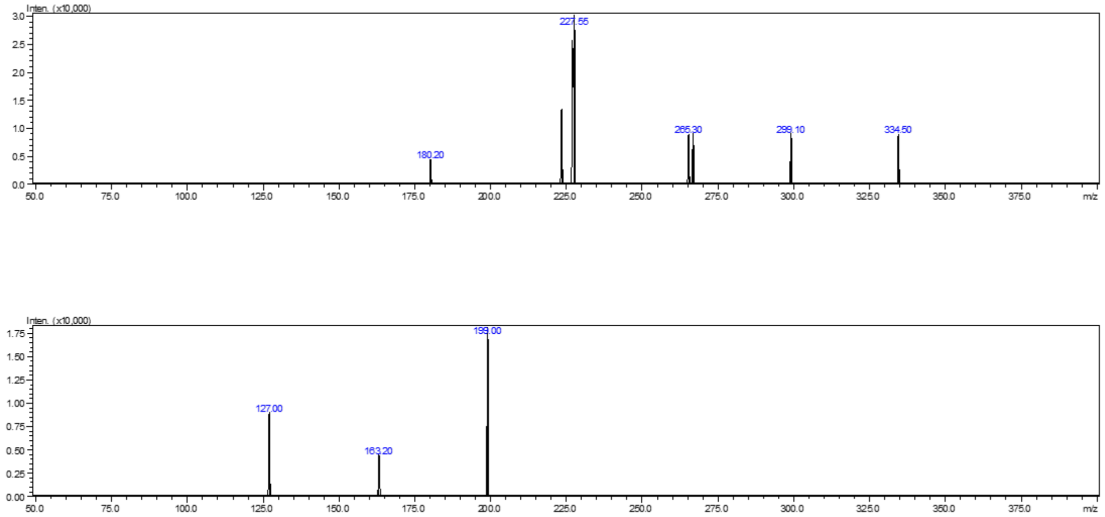

Figure S2h. QqQ-ESI-MS (top) and MS/MS (bottom) spectra of BPAF (m/z = 335) detected in pericardial fluid samples.

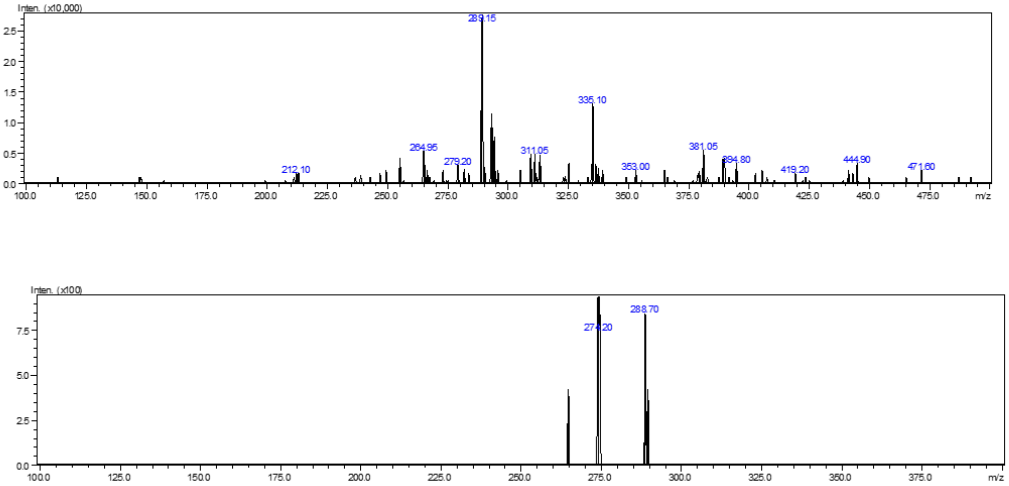

Figure S2i. QqQ-ESI-MS (top) and MS/MS (bottom) spectra of BPAP (*m*/*z* = 335) detected in pericardial fluid samples.

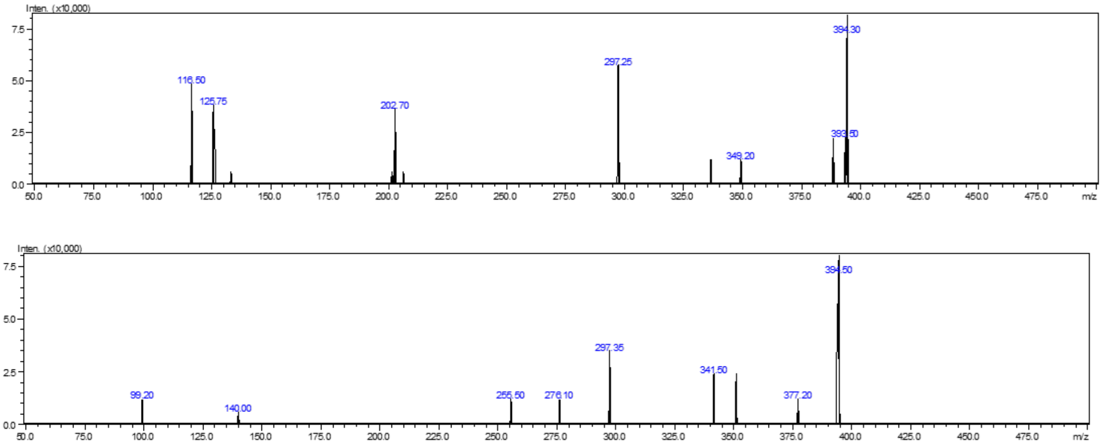

Figure S2j. QqQ-ESI-MS (top) and MS/MS (bottom) spectra of BADGE•$2H_2O$ (m/z = 394) detected in pericardial fluid samples.

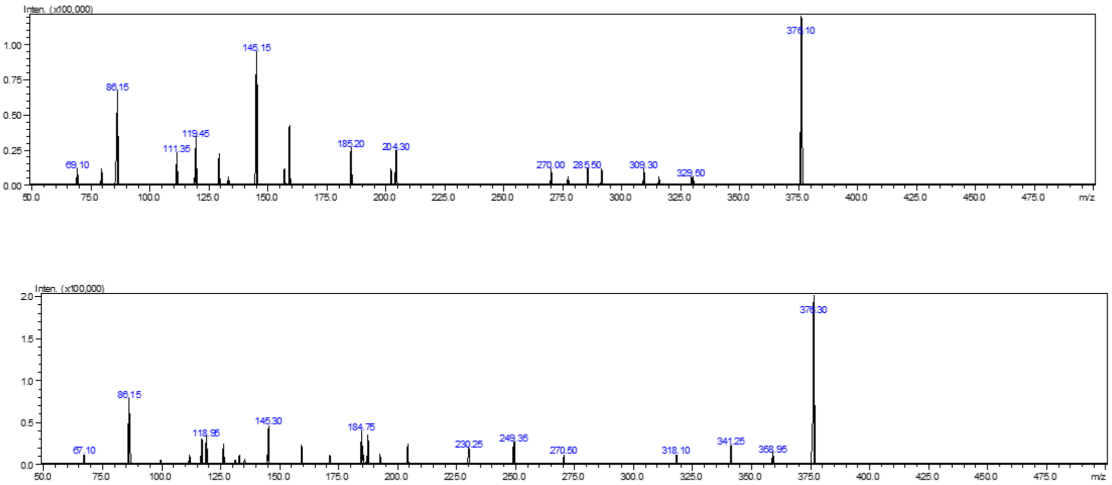

Figure S2k. QqQ-ESI-MS (top) and MS/MS (bottom) spectra of BADGE•H_2O (*m*/*z* = 376) detected in pericardial fluid samples.

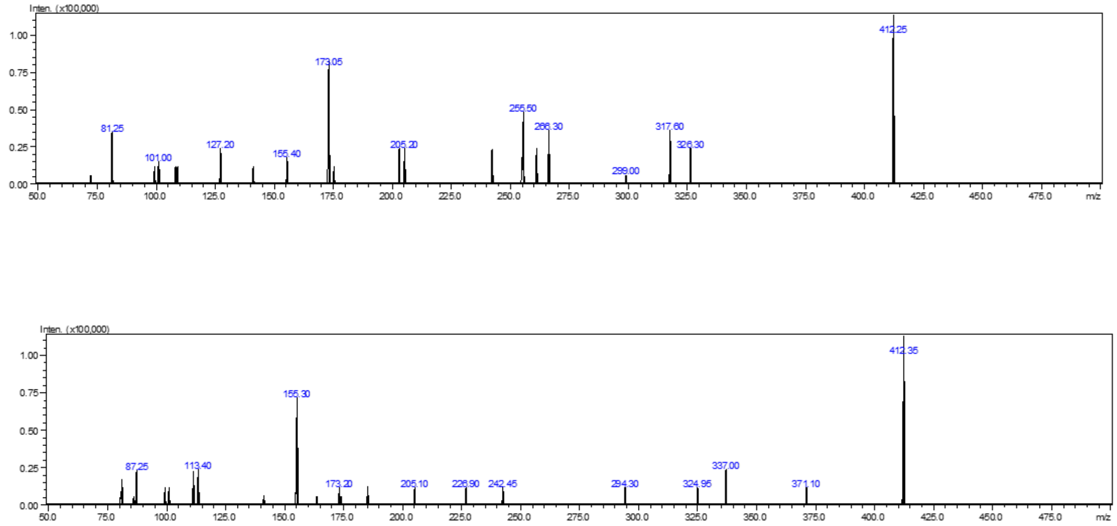

Figure S2l. QqQ-ESI-MS (top) and MS/MS (bottom) spectra of BADGE•H_2O•HCl (*m*/*z* = 412) detected in pericardial fluid samples.

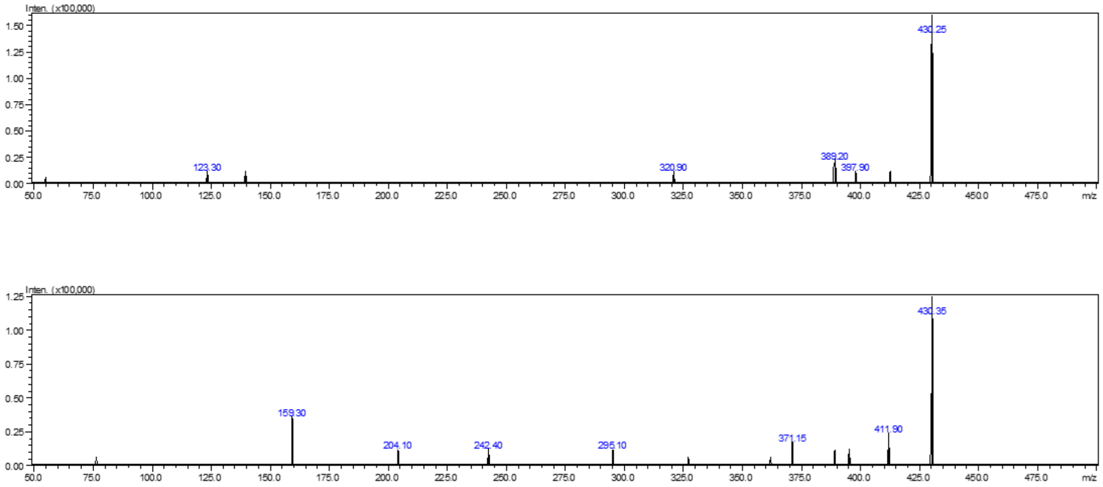

Figure S2m. QqQ-ESI-MS (top) and MS/MS (bottom) spectra of BADGE•2HCl (*m*/*z* = 430) detected in pericardial fluid samples.

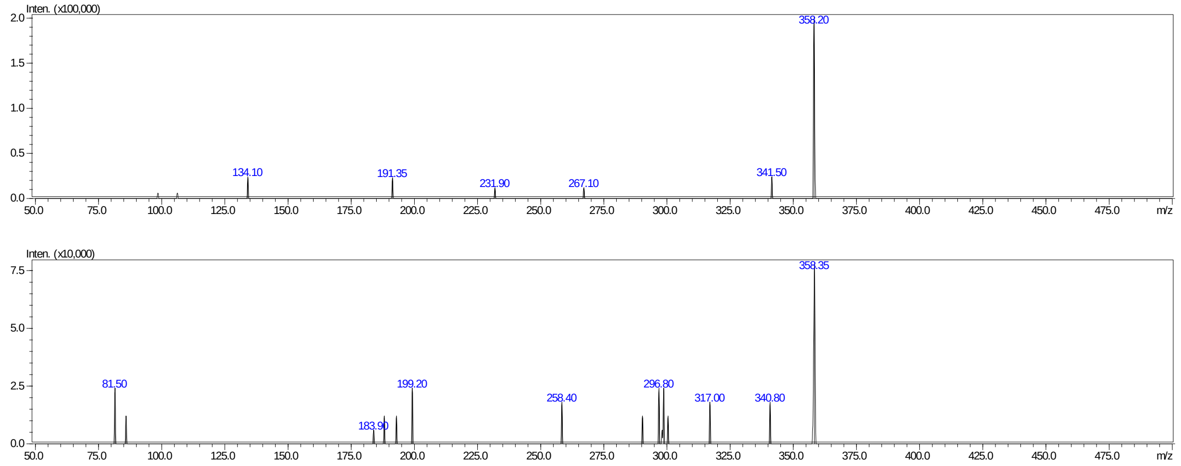

Figure S2n. QqQ-ESI-MS (top) and MS/MS (bottom) spectra of BADGE (*m*/*z* = 358) detected in pericardial fluid samples.

Figure S2. QqQ-ESI-MS (top) and MS/MS (bottom) spectra of following bisphenols residues detected in pericardial fluid samples: (**a**) BPS (*m*/*z* = 249), (**b**) BPF (*m*/*z* = 199), (**c**) BPE (*m*/*z* = 213), (**d**) BPA (*m*/*z* = 227), (**e**) BPB (*m*/*z* = 241), (**f**) BPP (*m*/*z* = 345), (**g**) BPZ (*m*/*z* = 267), (**h**) BPAF (*m*/*z* = 335), (**i**) BPAP (*m*/*z* = 335), (**j**) BADGE•2H_2O (*m*/*z* = 394), (**k**) BADGE•H_2O (*m*/*z* = 376), (**l**) BADGE•H_2O•HCl (*m*/*z* = 412), (**m**) BADGE•2HCl (*m*/*z* = 430), (**n**) BADGE (*m*/*z* = 358).

Table S1. Analysis of selected bisphenols in pericardial fluids collected from 19 patients with coronary artery diseases and undergoing coronary artery bypass surgery with the use of LC-ESI-QqQ.

No	BPA	BPS	BPB	BPF	BPP	BPE	BPZ	BPAP	BPAF	BADGE	BADGE•H_2O	BADGE•$2H_2O$	BADGE•H_2O•HCl	BADGE•2HCl
	m/z=227	*m/z*=249	*m/z*=241	*m/z*=199	*m/z*=345	*m/z*=213	*m/z*=267	*m/z*=335	*m/z*=335	*m/z*=358	*m/z*=376	*m/z*=394	*m/z*=412	*m/z*=430
1	1.47	nd	LOQ	LOQ	LOQ	1.22	0.68	0.25	nd	0.71	nd	LOQ	1.28	LOQ
2	1.79	nd	LOQ	LOQ	LOQ	1.25	0.61	0.30	nd	0.59	nd	LOQ	1.36	LOQ
3	1.39	nd	LOQ	LOQ	LOQ	1.15	LOQ	0.43	nd	0.14	nd	1.24	LOQ	LOQ
4	0.35	nd	nd	LOQ	nd	0.96	LOQ	0.43	nd	0.55	nd	nd	LOQ	nd
5	1.17	nd	LOQ	0.75	LOQ	1.44	LOQ	0.35	nd	0.44	nd	LOQ	0.75	LOQ
6	1.10	nd	LOQ	0.76	LOQ	1.10	0.61	0.28	nd	0.47	nd	1.27	0.76	LOQ
7	1.20	nd	0.68	0.84	LOQ	0.87	LOQ	0.30	nd	0.32	nd	1.27	LOQ	LOQ
8	1.22	nd	LOQ	0.71	LOQ	1.59	LOQ	LOQ	nd	0.48	nd	LOQ	LOQ	LOQ
9	0.94	nd	nd	nd	nd	1.03	0.61	LOQ	nd	0.46	nd	nd	nd	nd
10	1.16	nd	LOQ	nd	nd	0.73	LOQ	LOQ	nd	0.37	nd	LOQ	nd	nd
11	1.29	nd	0.72	nd	nd	1.09	LOQ	0.41	nd	0.81	nd	1.31	nd	nd
12	2.04	nd	LOQ	0.67	nd	0.97	nd	0.38	nd	0.51	nd	LOQ	0.67	nd
13	1.97	nd	LOQ	0.83	LOQ	1.82	nd	0.69	nd	0.71	nd	LOQ	LOQ	LOQ
14	1.78	nd	LOQ	LOQ	LOQ	1.05	LOQ	0.72	nd	0.79	nd	LOQ	LOQ	LOQ
15	1.83	nd	LOQ	0.82	LOQ	0.79	LOQ	0.69	nd	0.97	nd	LOQ	LOQ	LOQ
16	2.44	nd	LOQ	1.19	nd	1.13	LOQ	1.04	nd	0.73	nd	LOQ	1.19	nd
17	2.89	nd	LOQ	1.27	nd	1.25	0.59	1.17	nd	1.24	nd	LOQ	1.27	nd
18	3.11	nd	nd	1.76	LOQ	1.28	0.67	1.38	nd	1.15	nd	nd	1.76	LOQ
19	3.63	nd	nd	1.79	LOQ	1.84	0.58	1.93	nd	1.45	nd	nd	1.79	LOQ

Table S2. Validation of the HPLC-FLD method after DLLME method. Intra and inter-day accuracy (Recovery %) and precision (range (±)) and intra-laboratory reproducibility.

Bisphenols	Intra-day Accuracy[*1] % Recovery %									Mean recovery % [*2]		
	Day 1 Concentration (ng mL^{-1})			Day 2 Concentration (ng mL^{-1})			Day 3 Concentration (ng mL^{-1})					
	10	20	30	10	20	30	10	20	30	10	20	30
BADGE·2H_2O	62±2.1	58±1.4	63±2.3	60±2.0	60±1.8	62±2.3	63±2.2	59±1.6	64±1.3	61.7±2.1	59.0±1.6	63.0±2.0
BPF	81±3.4	79±4.3	99±4.0	82±2.4	79±4.9	98±4.2	84±3.0	80±4.9	98±3.8	82.3±2.9	79.3±4.7	98.3±4.0
BPE	93±1.8	79±2.2	86±4.0	92±1.9	80±3.2	85±4.6	93±2.2	81±4.2	84±4.5	92.6±2.0	80.0±3.2	85.0±4.4
BPA	92±2.5	92±2.2	89±2.4	92±2.6	91±2.6	89±2.8	92±2.8	91±3.0	90±2.6	92.0±2.6	91.3±2.6	89.3±2.6
BADGE·2HCl	98±4.3	90±4.5	89±4.2	97±3.5	89±4.1	89±4.7	96±4.1	88±4.4	91±4.0	97.0±4.0	89.0±4.3	89.7±4.4
BADGE	62±3.6	59±3.5	63±3.2	62±3.4	58±3.3	63±3.6	62±3.6	57±3.6	63±3.8	62.0±3.5	58.0±3.5	63.0±3.5
BPP	99±1.7	93±2.2	92±1.5	98±2.0	91±1.8	92±2.0	100±2.4	92±2.0	92±2.5	99.0±2.0	92.0±2.0	92.0±2.0

Bisphenols	Intra-laboratory reproducibility [*3]								
	Analyst 1 Concentration (ng mL^{-1})			Analyst 2 Concentration (ng mL^{-1})			Mean recovery % (n = 12)		
	10	20	30	10	20	30	10	20	30
BADGE·2H_2O	63±2.0	61±1.8	63±1.0	62±2.2	60±1.6	65±1.0	62.5±2.1	60.5±1.7	64.0±1.0
BPF	81±2.9	82±4.9	98±4.0	84±2.9	82±4.3	99±4.0	82.0±2.9	82.0±4.6	98.5±4.0
BPE	93±2.0	81±3.2	86±4.0	95±2.0	84±4.4	86±3.6	94.0±2.0	82.5±4.3	86.0±4.4
BPA	93±2.0	93±2.6	92±2.0	94±2.4	91±2.0	90±2.6	94.0±2.2	92.0±2.3	91.0±2.3
BADGE·2HCl	95±4.0	90±4.0	94±4.0	99±4.0	90±4.4	92±4.2	97.0±4.0	90.0±4.2	93.0±4.1
BADGE	64±3.8	60±3.5	67±3.5	66±3.5	62±3.5	64±3.6	64.0±3.7	61.0±3.5	65.0±3.5
BPP	100±2.0	94±1.2	96±2.0	99±2.0	95±1.5	94±2.0	99.5±2.0	93.0±1.4	92.0±2.0

[*1] – n = 6; [*2] – Mean recovery % from 18 samples analyzed in three different days (n = 6 for each day); [*3] – Mean recovery % from two analysts (n = 6 for each operator) and mean results (n=12);

Table S3. Validation of the HPLC-FLD method after DLLME method. Intra-day accuracy and precision for fortified level at 25 ng mL^{-1} (additionally results).

Bisphenols		Intra-day accuracy[*1] for concentration 25 ng mL^{-1}					
	Average concentration measured ng mL^{-1} Day 1 [*1]	Average concentration measured ng mL^{-1} Day 2 [*1]	Average concentration measured ng mL^{-1} Day 3 [*1]	Mean concentration measured ng mL^{-1} for three Days 1-3 [*2]	SD [*2]	SD / c_4 [*3]	RSD% [*2]
**BADGE·2H$_2$O**	16.35	16.15	16.55	16.35	0.16	0.165	0.98
**BPF**	24.63	24.93	24.85	24.80	0.05	0.052	0.20
**BPE**	21.25	21.55	21.95	21.58	0.29	0.299	1.34
**BPA**	22.51	22.60	22.65	22.59	0.03	0.031	0.13
**BADGE·2HCl**	22.49	22.30	22.51	22.43	0.05	0.052	0.22
**BADGE**	16.50	16.61	16.35	16.49	0.11	0.113	0.67
**BPP**	23.10	23.15	23.35	23.20	0.09	0.093	0.39

[*1] – n = 3

[*2] – Mean concentration, SD and RSD% from experiments during three days 1-3 (n = 9)

[*3] C_4 = 0,96931 (n = 9)

Extraction Recovery Studies, Accuracy and Precision

Mean recoveries were evaluated at three different concentration levels: 10 ng/mL, 20 ng/mL, and 30 ng/mL of the sample. Mean recovery values were obtained from six replicates for every spiking levels. Accuracy in all cases was expressed as percentage recovery of the analyte and calculated using the following formula:

$$\text{Recovery (\%)} = \frac{\textbf{average analyte concentration found in the sample}}{\textbf{analyte concentration added to the sample}} \times 100\% \qquad \text{(Eq. S1)}$$

The average analyte concentration found in the sample is regarding to the peak area of the analyte obtained after a procedure where sample was spiked before DLLME extraction.

The analyte concentration added to the sample is regarding to the peak area of analyte obtained after procedure where sample was spiked after DLLME extraction directly into vial.

Relative standard deviation was calculated as follows:

$$\text{RSD\%} = \frac{\textbf{standard deviation (SD) of the recovery}}{\textbf{mean recovery}} \times 100\% \qquad \text{(Eq. S2)}$$
